# Identification of Novel Loci Associated with Gastrointestinal Parasite Resistance in a Red Maasai x Dorper Backcross Population

**DOI:** 10.1371/journal.pone.0122797

**Published:** 2015-04-13

**Authors:** Magda Vieira Benavides, Tad S. Sonstegard, Stephen Kemp, John M. Mugambi, John P. Gibson, Robert Leyden Baker, Olivier Hanotte, Karen Marshall, Curtis Van Tassell

**Affiliations:** 1 LabEx USA, Embrapa, Brasília, DF, Brazil; 2 Animal Genomics & Improvement Laboratory, USDA/ARS/Beltsville Agricultural Research Center, Beltsville, MD, United States of America; 3 Animal Biosciences, The International Livestock Research Institute (ILRI), Nairobi, Kenya; 4 National Veterinary Research Centre, Kenya Agricultural Research Institute (KARI), Muguga, Kenya; 5 Centre for Genetic Analysis and Applications, University of New England, Armidale, NSW, Australia; 6 Whangamata, Waikato, New Zealand; 7 Medicine & Health Sciences, The University of Nottingham, Nottingham, United Kingdom

## Abstract

Gastrointestinal (GI) parasitic infection is the main health constraint for small ruminant production, causing loss of weight and/or death. Red Maasai sheep have adapted to a tropical environment where extreme parasite exposure is a constant, especially with highly pathogenic *Haemonchus contortus*. This breed has been reported to be resistant to gastrointestinal parasite infection, hence it is considered an invaluable resource to study associations between host genetics and resistance. The aim of this study was to identify polymorphisms strongly associated with host resistance in a double backcross population derived from Red Maasai and Dorper sheep using a SNP-based GWAS analysis. The animals that were genotyped represented the most resistant and susceptible individuals based on the tails of phenotypic distribution (10% each) for average faecal egg counts (AVFEC). AVFEC, packed cell volume (AVPCV), and live weight (AVLWT) were adjusted for fixed effects and co-variables, and an association analysis was run using EMMAX. Revised significance levels were calculated using 100,000 permutation tests. The top five significant SNP markers with - log10 p-values >3.794 were observed on five different chromosomes for AVFEC, and BLUPPf90/PostGSf90 results confirmed EMMAX significant regions for this trait. One of these regions included a cluster of significant SNP on chromosome (Chr) 6 not in linkage disequilibrium to each other. This genomic location contains annotated genes involved in cytokine signalling, haemostasis and mucus biosynthesis. Only one association detected on Chr 7 was significant for both AVPCV and AVLWT. The results generated here reveal candidate immune variants for genes involved in differential response to infection and provide additional SNP marker information that has potential to aid selection of resistance to gastrointestinal parasites in sheep of a similar genetic background to the double backcross population.

## Introduction

Gastrointestinal (GI) parasitic infections are amongst the main health constraint affecting grazing livestock worldwide [1,2]. Production levels can be greatly reduced by GI parasite infections, and, if left untreated, mortality rates can be high in severely affected animals. Lambs are born naïve and acquire immunity to gastrointestinal infections through continuous natural exposure to infective larvae while grazing. The combination of live weight loss and severe drop in packed cell volumes caused by haematophagous *Haemonchus contortus*, the most prevalent parasite in tropical and sub-tropical regions, are highly pathogenic to lambs, even in production regions relying on drug control.

Anthelmintic treatments are largely used to control parasite infections, however it has become evident that continuous drenching leads to massive selection pressure and reports point to parasite resistance against the main traditional chemical drugs commercially available [3,4]. Heavy reliance of livestock production on chemicals to control host parasites also have raised public opinion concerns due to the presence of drug residues in animal food products. Sustainable solutions to this problem have been under investigation for several decades, however alternatives for parasite control have proven slow and laborious.

In recent decades, breeding programmes based on phenotypic information have enabled farmers to identify sheep breeds and animals more resistant to parasitic diseases [5–9]. These have shown a steady reduction in faecal egg counts (FEC), with a concomitant decrease in number of drenches (http://www.csiro.au/files/files/p66p.pdf [10]; http://www.agresearch.co.nz/business/products/sheep-trait-recording/docs/WormFEC%20brochure.pdf [11]). However, challenge protocols are still needed in order to phenotype sheep as resistant or susceptible. This continuous validation of improved resistance is usually performed on weaned lambs to reduce generation intervals, but these procedures also suppress lamb production [12–14].

Many studies have attempted to identify genes or genetic variants responsible for parasite resistance in hopes of using this information to enable more effective animal breeding programmes, which result in less severe health stress on GI infected lambs. QTL and association studies have reported sheep gastrointestinal parasite resistance to every ovine chromosome (OAR), except for OAR 15, 17, 25, and X. Comparison of QTL and GWAS mapping results from these studies provide relatively strong evidence that the major histocompatibility complex (MHC) on OAR 20 and interferon gamma (IFN) on OAR 03 [15,16] are involved with host resistance. The first is an antigen presenter to T cells triggering the development of immunological responses [17] and the latter a strong Th_2_ response antagonist [18].

Because QTL studies rely on having individuals with extreme phenotypes for a desired trait, we chose to extend the mapping studies of a double backcross population derived from combining a composite high production breed (Dorper) with an indigenous breed (Red Maasai). The latter, has evolved under constant and heavy parasite exposure, and is known to better withstand *Haemonchus contortus* infections [18–21]. Previous QTL mapping results based on microsatellite-based analysis [22,23] detected host resistance for large genomic intervals on several chromosomes. In this extended study, we used genome-wide SNP genotyping, to validate these previous QTL positions in this flock, as well as, to identify new markers associated with parasite resistance.

## Material and Methods

### Population resource and phenotypes

Pedigrees, animal DNA samples and phenotypes used in this study were derived from a double backcross population of Red Maasai and Dorper from the International Livestock Research Institute (ILRI), which contained 1,081 individuals [24]. Briefly, the phenotype data includes average FEC (AVFEC) under natural challenge conditions, for the average of two measurements taken one day apart, packed cell volume (PCV) average (AVPCV), and live weight (LWT) average (AVLWT), traits later used for GWAS analyses. In addition, the PCV at the start of the challenge period (PCVST) and the decline in PCV from the start to the completion of the pasture challenge (PCVD) were calculated.

Phenotypic results from the Red Maasai x Dorper backcross sheep population have been extensively discussed in [24], and previous QTL study with microsatellite markers has been discussed in [23] (same phenotypic data, i.e. natural parasite challenge starting when 4-month old).

### Genotypes and GWAS analysis

Analysis of variance distributions of AVFEC, AVPCV and PCVD were used to identify the 10% most resistant and 10% most susceptible lambs for genotyping [23]. A subset of 371 lambs chosen for selective genotyping consisted of 192 resistant lambs, 173 susceptible lambs and 6 lambs resistant for one trait but susceptible for another, the 6 F1 rams (sires) and 11 Dorper and Red Maasai grandparents. DNA quality was checked by using Nanodrop (Thermo Scientific) and PicoGreen assay (Invitrogen), and 300 ng of DNA was processed using Illumina’s OvineSNP50 assay based on Infinium beadchip chemistry.

Marker genotype results for 54,241 SNP were filtered using PLINK. Markers were removed based on: minor allele frequency (MAF less than 1%), genotype call rates per marker (GCR less than 99.9%), and deviation from Hardy-Weinberg equilibrium for each SNP (p≥0.001). The final dataset for genome wide association (GWA) analyses contained 31,686 SNP marker genotypes for each animal (S1 Table).

AVFEC was analysed according to [23], after normalisation using Box-Cox transformation methods, prior to GWA analysis using an efficient mixed-model association (EMMA) algorithm after optimisation (eXpedited, hence EMMAX beta 07-Mar-2010 version) [25] to increase computational efficiency by using identical-by-state kinship matrix. To correct for population stratification, crossbred (¾ Red Maasai or ¾ Dorper), sire group (1–6), gender (male, female), lambing season (1–5), birth rank (single, multiple), age of dam (2–5+) were fitted as fixed effects and day of birth (within lambing season) as a linear co-variable in SAS (Proc mixed) analyses. Significant fixed effects and co-variable (P < 0.05) were used to generate phenotypic residuals to run EMMAX. Significance for each SNP marker—log10 p-values in EMMAX additive model was determined after 100,000 permutation runs [26], [27,28]. EMMAX was not designed to analyse dominance effects therefore only additive effects were calculated.

Additionally, the PostGSf90 module of BLUPf90 package [29] was used to analyse results at a sliding window of 100 markers at a time in an attempt to account for linkage disequilibrium (LD) among SNPs, a feature not available in EMMAX. This software package also allows fitting fixed and random effects and co-variables in the model. The main focus of PostGSf90 is not to estimate—log10 p-values, but instead SNP solutions and the variance explained by specific or groups of markers, as in the case of sliding window option, using a relationship matrix based on pedigree and genomic information inverted by algorithms described in [29]. Revised significance levels were calculated fitting the same model and also running 100,000 permutation tests for the PostGSf90 module.

### Post GWA analyses

Most of the literature on sheep resistance to gastrointestinal parasites is based on microsatellite markers, their correspondent base pair (bp) positions were retrieved for comparison of position effects found using the OvineSNP50, with either the help of Sheep Genome Browser Oarv1.0 (http://www.livestockgenomics.csiro.au/cgi-bin/gbrowse/oar1.0/) or specific primer sequence aligned to the Baylor Btau_4.6.1/bosTau7 (Oct 2011) cow genome assembly using BLAT (http://genome.ucsc.edu/cgi-bin/hgBlat?command=start).

The locations of significant SNP markers were compared to human RefSeq data, using ±1Mbp as flanking regions. The list of human RefSeq IDs was transformed to DAVID IDs (Database for Annotation, Visualization and Integrated Discovery v6.7; http://david.abcc.ncifcrf.gov) [30] by using the gene accession conversion tool and analysing functional annotation clustering. Using this same approach to compare to bovine RefSeq data resulted in mostly XM and XR transcripts (predicted mRNA and non-coding RNA information, respectively). Therefore a second attempt was made by searching for homology of the significant SNP marker oligo sequences in the bovine genome using the blat option of the UCSC genome browser (http://genome.ucsc.edu/cgi-bin/hgBlat?command=start) to find homologies in the *Bos taurus* genomic DNA and search for genes annotated at flanking regions of the SNPs of interest. Having this information, biological pathways of each gene were analysed using the FLink tool (http://www.ncbi.nlm.nih.gov/Structure/biosystems/docs/biosystems_about.html).

In summary, the bioinformatics workflow used for SNP data analysis was:

(a) GWAS analyses:
(a.1) Quality control (GenomeStudio);
(a.2) Fixed effects for phenotypic data (SAS);
(a.3) PLINK filters;
(a.4) GWAS analyses (EMMAx beta 07-Mar-2010 version and BLUPf90/PostGSf90 version 1.28);
(b) Post GWAS analyses:
(b.1) Search for genes close to SNP markers at Sheep Genome Browser Oarv3.0 (www.livestockgenomics.csiro.au/sheep/oar3.1.php);
(b.2) Comparisons to human RefSeq data (http://david.abcc.ncifcrf.go);
(b.3) Blat search for homology in the UCSC Bovine Genome Browser (http://genome.ucsc.edu/cgi-bin/hgBlat?command=start);
(b.4) Search for biological pathways using FLink tool (http://www.ncbi.nlm.nih.gov/Structure/biosystems/docs/biosystems_about.html);
(b.5) Expression of candidate genes in immune response tissues (ftp://ftp.ensembl.org/pub/release-74/bam/ovis_aries/genebuild/).

## Results

### Descriptive statistics

Descriptive statistics of phenotypic traits for the selectively genotyped Red Maasai x Dorper backcrossed sheep are presented in Table 1. As expected, more resistant animals showed lower (back-transformed) faecal egg counts (3.93 times lower than susceptible animals), had 52% higher levels of packed cell volume, and were 12% heavier than susceptible animals. Resistant animals started the experimental challenge period with PCV levels lower than susceptible individuals. The susceptible animals in this study showed severe anaemia and loss of weight, typical hallmarks of *Haemonchus contortus* infections.

**Table 1:** Descriptive statistics of Red Maasai x Dorper backcrossed sheep selected phenotypes.

		10% more resistant	10% more susceptible
Trait	Trait description	Mean (±Std. err.)	Mean (±Std. err.)
AVPCV	Average (AV) final PCV (%)	28.39±0.21 ^a^	18.57±0.36 ^b^
PCV_ST	PCV at the start of the pasture challenge	34.78±0.30 ^a^	35.25±0.34 ^b^
PCVD	Decline in PCV from the start to the completion of the pasture challenge (PCVD)	6.39±0.31 ^b^	14.68±0.45 ^a^
AVLWT	Average (AV) final LWT (kg)	19.18±0.33 ^a^	17.00±0.32 ^b^
FEC	Average faecal egg count (eggs per gram) from measurements on two consecutive days at the end of the pasture (P) challenge period	5,941±335.29 ^b^	23,328±889.55 ^a^
LFEC	Log transformation of FEC values	3.64±0.026 ^b^	4.31±0.018 ^a^
AVFEC	Box-Cox transformation of FEC values	25.31±0.377 ^b^	37.02±0.377 ^a^

Traits: Packed cell volume (AVPCV), starting packed cell volume (PCV_ST), decline in packed cell volume (%) (PCVD), liveweight (AVLWT), non-transformed faecal egg counts (FEC), log-transformed FEC (LFEC), Box-Cox transformed FEC (AVFEC)

Letters correspond to Tukey HSD test significance using α = 0.05.

Among the significant fixed effects for AVPCV were crossbred and lambing season (P = 0.0014 and 0.0032, respectively), for AVLWT were gender, crossbred, lambing season, birth rank (P = 0.0066, < 0.0001, < 0.0001, and <0.0001, respectively), and the co-variable day of birth (P = 0.0001), and for AVFEC were crossbred and lambing season (P = 0.0004 and < 0.0001, respectively).

### GWAS identifies novel chromosomal regions linked to phenotypic resistance traits

**Fig 1:**
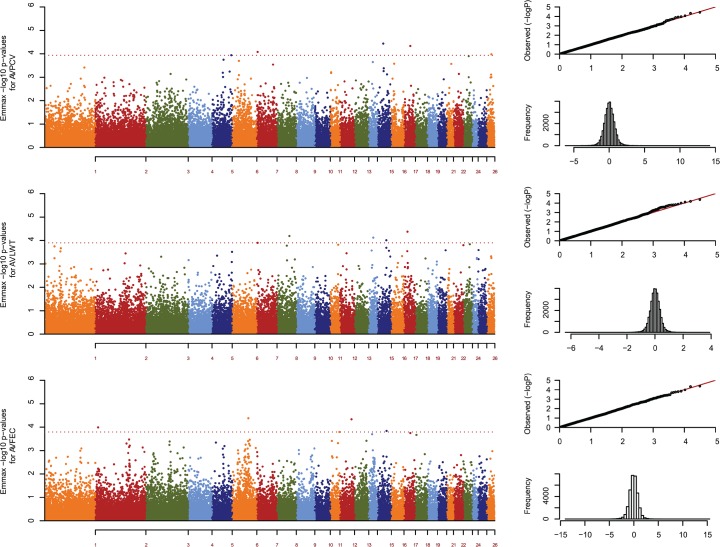
Manhattan plot with GWAS for AVPCV, AVLWT, and AVFEC. QQ plots of observed −log10 p-values against expected values (top) and histogram of SNP effects (bottom) on the right hand side. Dashed red line for AVPCV corresponds to the lowest −log10 p-value amongst relevant SNPs (3.936).Dashed red line for AVLWT corresponds to the lowest −log10 p-value amongst relevant SNPs (3.898).Dashed red line for AVFEC corresponds to the lowest −log10 p-value amongst relevant SNPs (3.794).

EMMAX's Manhattan plots for all analysed traits are presented in Fig 1. In order to identify significant SNP markers, permutations were used as a first threshold and it was observed that, for AVFEC, 0.7% of the SNP markers had reached significance levels (221/31,686 markers). The respective figures for AVPCV were 0.62% (198/31,686), and 0.56% (177/31,686) for AVLWT.

Because of the high number of significant markers for AVFEC after permutation results, a second threshold based on top—log10 P-values was used to lower the number of markers down, arbitrarily, to the top five most relevant markers (- log10 p-value ≥3.794). Amongst them were three novel autosomal regions identified in the Red Maasai x Dorper population, and not previously reported in other parasite resistance mapping studies of other sheep breeds. These regions were found on OAR2 (15 Mbp), OAR11 (58 Mbp), and OAR15 (54 Mbp).

The existence of clusters of significant markers, though showing lower—log10 p-values than the second threshold chosen for the relevant markers, was also analysed. The outcome of that exercise resulted in 30 markers arranged in nine clusters on chromosomes 1, 2, 3, and 6. Two novel regions were found on OAR2 (162–163Mpb), and OAR3 (44 Mbp). The list of relevant SNP markers and marker clusters are shown in Table 2. Red Maasai x Dorper GWAS results compared to other QTL, candidate gene and GWA studies are displayed in S1 Fig.

**Table 2:** AVPCV, AVLWT, and AVFEC relevant SNP markers: top −log_10_ p-value and cluster markers.

Top SNP marker	Significant SNP in clusters	OAR/ cluster	position (bp)	EMMAX −log10 pvalues	EMMAX beta estimate	EMMAX permuted −log10 pvalues	BLUPf90 100 adjacent SNP effect (±var)
**AVPCV**							
OAR5_111342555		5	102,275,828	3.9365	2.0046	2.2098	4.160
OAR7_4206430		7	4,455,015	4.0752	-2.0542	2.3039	4.112
OAR15_35337227		15	33,590,990	4.4336	-2.1156	2.3145	3.873
OAR17_42673146		17	39,494,633	4.3398	4.4156	2.2613	1.896
OAR26_28808248		26	24,839,177	3.9756	-2.3858	2.0221	3.500
**AVLWT**							
OAR7_4206430		7	4,455,015	3.898	0.956	2.229	1.950
OAR8_63456328		8	58,938,494	4.188	-1.270	2.216	1.649
OAR14_32154219		14	30,920,062	4.118	-1.060	2.253	3.329
OAR15_58595710		15	53,451,424	4.005	-1.035	2.213	2.274
OAR17_24034449		17	21,658,699	4.374	1.027	2.256	3.480
**AVFEC**							
	OAR1_25121292	1/1	24,901,207	2.526	2.295	2.271	2.722
	OAR1_28678534	1/1	28,221,350	2.574	1.869	2.139	4.387
	OAR1_208747910	1/2	193,348,951	2.989	-3.073	2.222	2.510
	OAR1_208772113	1/2	193,374,188	2.527	-3.792	2.175	1.694
OAR2_14765360		2	15,322,091	3.990	2.372	2.268	3.475
	OAR2_172448710	2/1	162,765,073	3.472	-2.311	2.163	3.852
	OAR2_172603349	2/1	162,861,327	3.152	-2.196	1.945	3.428
	OAR2_173435842	2/1	163,790,869	2.325	-2.243	2.240	2.174
	OAR2_173670917	2/2	164,035,357	2.597	-2.243	2.156	2.513
	OAR2_174009207	2/2	164,375,300	2.442	1.765	2.232	4.575
	OAR2_174955986	2/2	165,310,919	2.371	-2.006	2.126	2.509
	OAR2_175122529	2/2	165,486,783	3.210	2.078	2.274	3.798
	OAR3_47667848	3/1	44,631,009	2.553	-1.933	2.216	2.959
	OAR3_47755179	3/1	44,725,366	2.423	-1.934	2.217	2.154
	OAR3_132008863	3/2	123,851,264	3.241	2.124	2.072	3.782
	OAR3_134032158	3/2	125,666,883	3.386	5.259	2.149	1.873
	s53138	3/2	130,182,335	2.956	-2.253	2.297	2.727
	OAR3_138897387	3/2	130,230,174	2.358	-2.215	1.937	2.177
	OAR6_61706883	6/1	55,942,491	2.694	-2.802	2.193	1.850
	OAR6_61732861	6/1	55,961,701	2.590	-2.761	2.177	1.787
	OAR6_62015468	6/1	56,257,759	2.983	-3.477	2.239	1.381
	s07402	6/1	59,788,134	3.056	2.252	2.302	3.615
	OAR6_66035942	6/1	60,015,208	2.716	-1.968	2.093	3.465
	s18431	6/1	60,135,240	2.500	-2.675	2.217	2.530
	s54665	6/1	60,473,797	2.745	-2.542	2.235	2.142
	OAR6_67918660	6/1	61,786,495	3.248	-2.371	2.181	3.384
	OAR6_68740207	6/1	62,601,182	2.523	-3.192	2.240	1.829
	OAR6_80892986	6/2	74,083,963	3.401	-2.607	2.188	3.177
	OAR6_80909611	6/2	74,098,684	2.853	1.923	2.164	3.226
OAR6_81718546		6	74,867,312	4.382	-2.480	2.140	3.951
	OAR6_85263669	6/3	78,111,034	3.290	-2.619	2.256	3.946
	OAR6_85367529	6/3	78,194,522	2.491	-2.732	2.224	2.913
OAR11_62887032		11	58,151,039	3.794	2.387	2.110	3.980
OAR12_69606944		12	63,088,234	4.339	-3.020	2.261	3.519
OAR15_59871543		15	54,529,249	3.836	2.728	2.179	3.544

The highest—log10 p-value SNP marker, OAR6_81718546, located at 74.86 Mbp was not in linkage disequilibrium with nearby SNP markers at 73.1–74.08 or 78.11–78.19 Mbp, hence it suggests the possibility that up to three QTLs are located within 73.1–78.19 Mbp region of OAR6 and with a fourth QTL nearby at 55.9–62.6Mbp. Further confirmation that OAR6_81718546 was not linked to neighbouring markers was obtained by removing this marker from the EMMAX genotype file. Output results showed that only the next upstream SNP marker changed its—log10 p-value, thus confirming its independence from other markers in the region. This SNP marker explains 3.95% of the variance observed for FEC, the second highest variance recorded for this study, measured by BLUPf90/PostGSF90 version 1.28 (Table 2).

**Fig 2:**
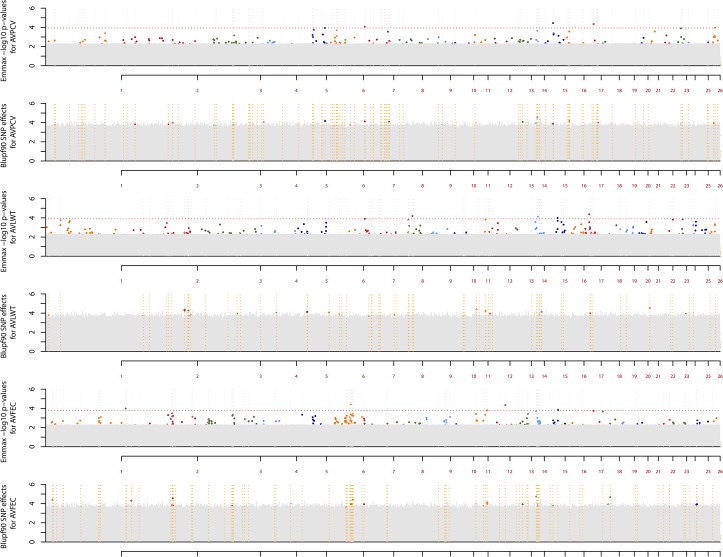
Manhattan plot for AVPCV, AVLWT, and AVFEC using EMMAX (permutation results in grey) and BLUPf90/PostGSf90 (permutation results in grey). Horizontal dashed lines in orange represent significant SNP markers in the BLUPf90/PostGSf90 analysis. Dashed red line for AVPCV corresponds to the lowest −log10 p-value amongst relevant SNPs (3.936).Dashed red line for AVLWT corresponds to the lowest −log10 p-value amongst relevant SNPs (3.898).Dashed red line for AVFEC corresponds to the lowest −log10 p-value amongst relevant SNPs (3.794).

The 74.8 Mbp chromosomal region and three clusters located at 55.9–62.6, 73.1–74.08, and 78.11–78.19 Mpb on OAR6 also coincided with BLUPf90/PostGSf90 significant results (60 Mbp to 85 Mbp) and accounted for the second highest variance observed for AVFEC (Fig 2). BLUPf90/PostGSf90's SNP effects for relevant SNP markers can be also seen on Table 2.

As for the other traits, four significant markers (OAR5_111342555, OAR7_4206430, OAR15_35337227, and OAR17_42673146), and all significant markers (OAR7_4206430, OAR8_63456328, OAR14_32154219, OAR15_58595710, and OAR17_24034449), for AVPCV and AVLWT, respectively, were located in chromosomal regions not previously reported in other QTL and GWA studies. The OAR7_4206430 marker was significant for both AVPCV and AVLWT.

**Fig 3:**
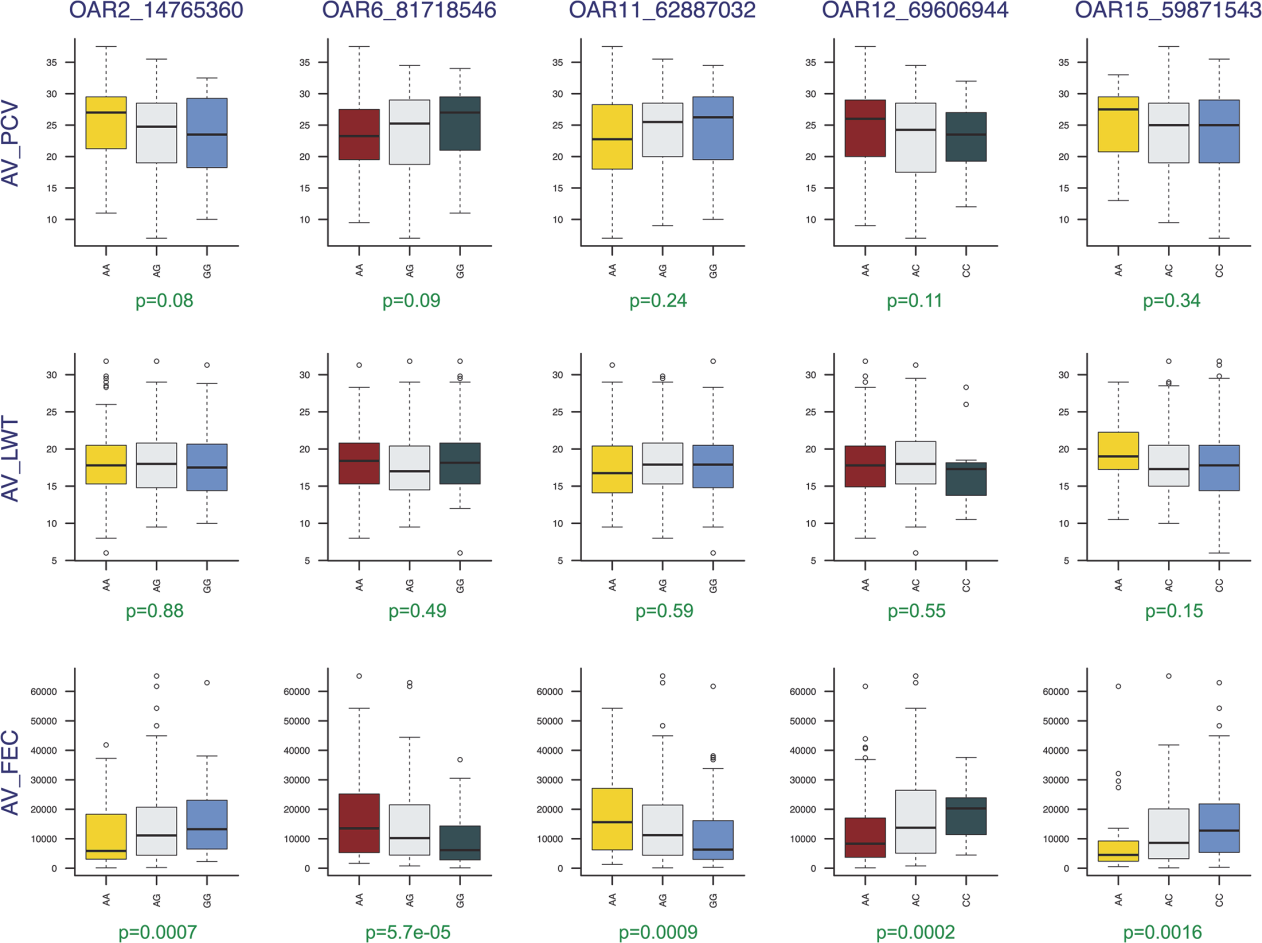
Genotype effects of AVFEC top SNP markers on packed cell volume and live weight.

The set of five top SNP markers explained 2.17%, 3.7% and 2.33% of the phenotypic variation for AVPCV, AVLWT and AVFEC, respectively. As far as phenotypes are concerned, top—log10 p-value SNP marker genotypes associated to AVFEC did not cause significant differences on packed cell volume or live weight (Fig 3).

**Fig 4:**
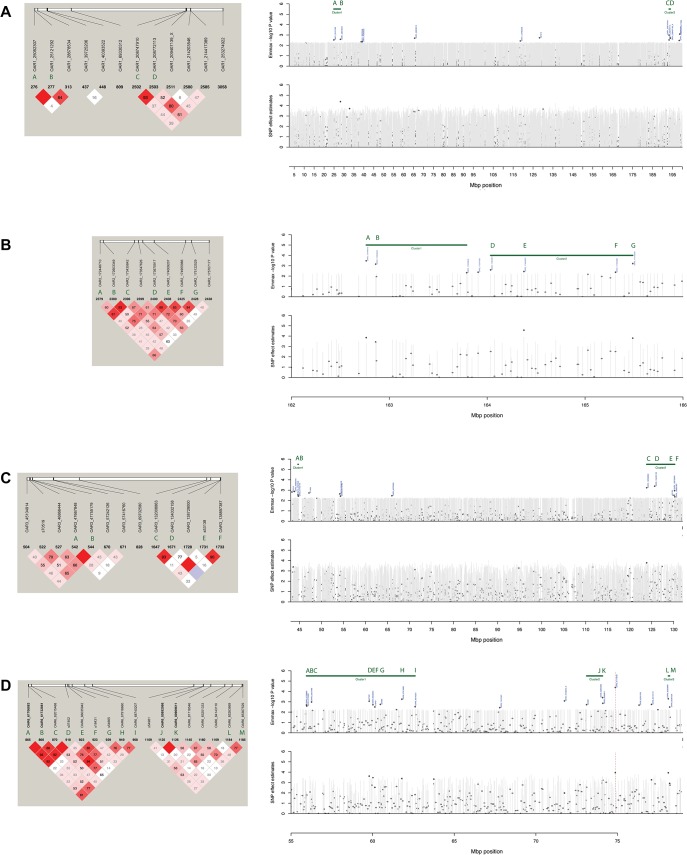
Top SNP markers and cluster positions on sheep chromosomes (OAR) 01, 02, 03, and 06 (a-d). Dashed red line corresponds to the SNP with the highest −log10 p-value.

To address the question of how results on EMMAX—which calculates SNP marker-phenotype association by testing one marker at a time—would compare to BLUPf90/PostGSf90—which allows setting "moving windows" to calculate the variance explained by *n* adjacent markers in an attempt to account for linkage disequilibrium—we ran an analysis on BLUPf90/PostGSf90 using 100 adjacent markers and results showed that 0.39% (124/31,686), 0.71% (226/31,686), and 0.33% (105/31,686) of markers were found to be significant for AVPCV, AVLWT, and AVFEC, respectively. With SNP effects ranging from 4.11 to 1.89 for AVPCV, 4.29 to 0.28 for AVLWT, and 3.97 to 3.47 for AVFEC. Fig 2 shows Manhattan plots for AVPCV, AVLWT and AVFEC, with non-permuted and permuted EMMAX—log p-value and BLUPf90 SNP effect results. Individual and cluster SNP marker locations and their linkage disequilibrium with significant flanking markers are presented in Fig 4. Comparing AVFEC results from EMMAX and BLUPf90/PostGSf90 packages, all significant markers found in EMMAX were also significant for BLUPf90/PostGSf90, except for OAR2_14765360 (Fig 2).

### Identification of genes underlying significant GWA results

Probe sequences of SNP markers from Table 2 aligned to 84 mRNA (NM) and non-coding RNA (NR) RefSeq sequences located at ±1Mbp distance using DAVID. These identified genes belonging to 35 biological processes (P-value < 0.05; S2 Table) from three functional annotation clusters involved in protein catabolism and proteolysis, purine ribonucleotide biosynthesis and purine nucleotide binding. These processes are associated with several pathways, which makes it difficult to single out the most relevant pathways affecting host response to infection. BLAT searches on bovine genome browser (*Bos taurus* Baylor Btau_4.6.1/bosTau7 assembly, Oct 2011) identified 59 of the 84 genes uncovered by DAVID.

FLink, from NCBI, was used to associate genes to biological pathways. Results from this analysis (S3 Table) showed key genes in mucus biosynthesis (GALNT4 and MUC15), cytokine signalling (SOCS2, UBE2N, EPS15, TLR10, KIT, LAMC1, and PDGFRA), and haemostasis (ATP2B1 and LRP8), matching to important pathways involved in host immune response against parasites.

In an effort to provide further evidence that the genes found closely located to the significant SNP association could be implicated in parasite resistance, we investigated the expression of the positional candidate genes in select tissues of critical importance to the immune response on sheep BAM files (ftp://ftp.ensembl.org/pub/release-74/bam/ovis_aries/genebuild/) and results show that EPS15, LRP8, GALNT4, and ATP2B1 genes were expressed in adult (ewe) and young (lamb) abomasa, mesenteric lymph nodes (MLN), and Peyer's patches (Table 3). MUC15 also showed the same pattern of expression as the other positional candidates except it was not expressed in lamb abomasum.

**Table 3:** Expression of genes close to significant SNPs in gastrointestinal tissues of adult and young sheep (abomasum, mesenteric lymph node (MLN), and Peyer's patch (PP)), closest gene with its position on the sheep genome browser, and distance from SNP polymorphism.

Gene name	OAR	Ewe abom.	Ewe MLN	Ewe PP	Lamb abom	Lamb MLN	Lamb PP	OAR position begin (bp)	OAR position end (bp)
**EPS15**	1	x	x	x	x	x	x	25,557,772	25,839,810
**LRP8**	1	x	x	x	x	x	x	27,524,283	27,600,322
**GALNT4**	3	x	x	x	x	x	x	125,769,422	125,825,227
**ATP2B1**	3	x	x	x	x	x	x	125,851,175	125,921,707
**SOCS2**	3	x	x	x	x	x	x	129,720,516	129,722,511
**UBE2N**	3	x	x	x	x	x	x	129,578,500	129,599,832
**SOX9**	11	x	x	x	x	x	x	57,796,760	57,800,093
**LAMC1**	12	x	x	x	x	x	x	62,163,057	62,284,313
**MUC15**	15	x	x	x		x	x	55,406,106	55,411,497

Livestock Genomics sheep genome browser v3.1

## Discussion

To identify some of the gene variants involved in differential immune response, many studies have attempted to find genetic markers associated to sheep gastrointestinal resistance (S4 Table) and there is mounting evidence that faecal egg count (FEC), the most indirect trait studied, is polygenic and not influenced by genes with major effects. The results from our Red Maasai x Dorper double backcross population reinforce previous findings that there are indeed many significant SNP markers associated to AVFEC (0.33% of the markers (105/31,686)). This result was not surprising and it strongly supports a multi-genic effect of host resistance to parasites. The agreement between BLUPf90/PostGSf90 and EMMAX results was also important because it provided support for using EMMAX as choice of GWAS software package. As a practical result, sheep breeding programmes could use the panel of relevant markers (and clusters) from Table 2 to select for more resistant Red Maasai and Dorper sheep.

While we concede that in an F2 population there are long stretches of genome between recombination of the breed specific chromosomes, there are still markers segregating within these two breeds that should help localise loci within breeds. Due to fiscal constraints, we were unable to expand this study to a greater number of animals, limiting to the 10% most resistant and 10% most susceptible lambs.

### AVFEC marker results agreement with literature

Overall, our GWAS results were similar to several results from literature, and confirm previous QTL mapping in this population. However, four new autosomal regions: OAR2 (15 Mbp), OAR3 (44 Mbp), OAR11 (58 Mbp), and OAR15 (54 Mbp) were revealed using the comprehensive Ovine SNP assay. Novel association results would be expected because previous efforts have not used indigenous breeds for QTL detection, with the exception of Martinique Black Belly sheep, and our population has both the Red Maasai and Blackhead Persian (Somali) (Dorper is a Somali x Dorset Horn synthetic breed). These breeds have undergone adaptation to tropical environmental conditions with much less exposure to artificial selection for production. As a consequence, indigenous animals might have developed mechanisms to tackle heavy parasite burdens over time, typical in such climates, differently from the commercial breeds discussed earlier.

The highest—log10 p-value SNP marker, OAR6_81718546, also maps to the same region reported previously [31], [32], [33], and [23]. The latter analysed microsatellite markers in this same resource population thus providing support that this genomic region affects immune response. Concomitantly, it also maps to the 60–80Mbp region identified by BLUPf90/PostGSf90 analyses as having significant SNP effects for AVFEC (Fig 2 and S1 Fig).

In addition, results from [23] reported a 5% genome-wide significant QTL in OAR6 BM1329 (45.0 cM)—BMS360 (80.8 cM), a wide genomic region that includes OAR6_81718546's location. On the other hand, [23] findings in OAR3 did not match results found here (S1 Fig). SNP analyses might disagree with microsatellite studies, however in our case three out of four genomic regions showed consistent results with previous studies [23].

As for the other significant markers, a cluster at OAR01 ("cluster 1") 24.9–28.2 Mbp was comparable to the results of [34], and "cluster 2" at OAR01 193.34–193.37 Mbp overlapped with results from [35] and [36] (Fig 4). Relevant markers in OAR3 ("cluster 2" at 123.8–130.2 Mbp) mapped within ranges previously reported [37–38], nonetheless in a genomic region not in LD with interferon gamma.

The OAR12_69606944 marker region differs from previous findings, as it distantly maps in between results earlier reported [38] where a QTL in the BM719—HUJ625 region had a LOD score of 2.7 with second *Trichostrongylus colubriformis* infection in Merino sheep, and further upstream from the s23035.1—OAR12_56589339.1, located at 51.1Mbp, described in INRA 401 x Martinique Black Belly backcross lambs infected with *Haemonchus contortus* [39].

S4 Table shows how extensive FEC genetic association results are and it is unlikely these will completely agree across most sheep breeds. The reason to expect so many marginally significant results would derive from the nature of the immune response, where host resistance is dependent on many individual immunological responses [40–44], as well as peristalsis [45–47], which plays important role on parasite expulsion. Both mechanisms are characterised by biochemical cascade of events that influence humoral immunity and effector cells, and makes host response a multifaceted trait involving a plethora of genes. Adding to this is the way research evaluates sheep immune response in the field, by increasing the number of factors related to sheep breeds and their different allelic frequencies, different experimental approaches taken to evaluate sheep immune response including infection protocols (natural *vs*. artificial), the species of gastrointestinal parasite infection (*Haemonchus contortus*, *Teladorsagia circumcincta*, and *Trichostrongylus colubriformis*) and their distinct immunological responses, phenotypic traits measured, environmental conditions under which the animals were raised (tropical *vs*. temperate, dry *vs*. humid climates), experimental designs (QTL experimental design *vs*. population studies), and statistical methods for data analysis add great complexity to interpret the results and difficulty in finding general agreement among references.

Another important outcome from the Red Maasai x Dorper results was that top—log10 p-value SNP marker genotypes associated to AVFEC did not affect packed cell volume, nor live weight (Fig 3), as selecting animals for more than one trait at a time is usual in breeding programmes. Impacts of selection for FEC on live weight have been controversial. Genetic correlation estimates between FEC and live weight vary largely, ranging from 0.11 in Romney [48], 0 to -0.3 in Romney [49,50], Merino [51] and Texel [52] to -0.6 to -0.8 in Polish long-wool [53] and Scottish Blackface sheep [54], however, estimates showed low FEC Red Maasai x Dorper sheep would not be expected to have detrimental effects on live weight.

### Relevant markers associated to packed cell volume and live weight

Reduction in packed cell volume is a sequelae of *Haemonchus contortus* infection and the inability of hosts to replenish red blood cell levels can lead to death. GWA studies for packed cell volume have been less extensively reported than for faecal egg count. Of the five top—log10 p-value markers for AVPCV, OAR5_111342555, and OAR15_35337227 are located close to those reported in [39] (OAR5_100699982.1—DU183841_402.1, and OAR15_40719719.1—OAR15_40926306.1), respectively, and OAR26_28808248 is located within ranges previously reported by [22]. Additional significant SNP markers confirm results from [39] at chromosomes 5 and 15, and those from [22] at chromosome 26. AVPCV associations to OAR7_4206430 and OAR17_42673146 are novel findings.

Other detrimental effect of helminthic infections is live weight loss. All top—log10 p-values for AVLWT were located in chromosomal regions previously undetected by other studies. Significant markers for live weight gain have been reported at OAR 1 and 3 by [55] and [56], respectively, who reported live weight QTL on Charollais, and Suffolk and Texel sheep at similar age (20-week old). Significant markers in OAR20 were described in a previous study [57] using 16-week old lambs, similar ages to the Red Maasai x Dorper lambs when phenotyped.

### Candidate genes located close to AVFEC markers

Finding important genes behind the biological mechanisms of a resistant individual have long been sought by the sheep industry. Genome-wide association studies not only uncover relevant markers associated to a specific productive trait, allowing their use for breeding programmes, but also permit the identification of genes that contribute to the expression of that phenotype.

#### (a) Relevant markers

Surprisingly, there was no gene information near our top—log10 p-value SNP, OAR6_81718546. The closest gene of interest to this marker was platelet-derived growth factor receptor alpha polypeptide (PDGFRA), which had been reported earlier [38], however the distance of this gene to our top SNP marker is 5 Mbp, therefore unlikely to be in linkage disequilibrium. Likewise, the closest gene to OAR2_14765360 was Krüppel-like factor 4 (gut) (KLF4), at 7.9 Mbp distance for this marker. The existence of genomic areas without gene information might be due to lack of sheep genome annotation as the reference genome assembly is still in a state of ongoing improvement [58].

OAR11_62887032 was located at 350,946 bp distance from SRY sex determining region Y-box 9 (SOX9) (Table 3), a transcription factor involved in embryonic and normal skeletal development, and also found to be significantly expressed during *Haemonchus contortus* infection in sheep [59].

OAR12_69606944 is positioned at 803,921 bp distance from laminin gamma-1 chain precursor (LAMC1). Laminins have been implicated in cell adhesion, differentiation, migration, signalling, and metastasis. It has been suggested that protozoan (*Trypanosoma cruzi*) surface proteins can interact with laminins and modulate host response in order to improve transport through host membrane barriers [60].

OAR15_59871543 was found at 876,857bp distance from mucin 15 cell surface associated (MUC15). Mucins encode epithelial glycoproteins that compound the mucus on the surface of epithelial tissues, acting as a physical barrier against viral, bacterial, fungal pathogens [61–62], and parasites [63–65]. *Trichinella spiralis* and *Nippostrongylus brasiliensis* were found covered by mucus before expulsion [65–67] and parasite motility and feeding being hampered when larvae were co-cultured with extracted intestinal mucus [66].

#### (b) Marker clusters

Of the SNP markers found in clusters, the most interesting results were genes found in proximity to marker s53138: suppressor of cytokine signalling (SOCS2), at 459,824bp, and Ubiquitin conjugating enzyme E2N (UBE2N), at 582,503bp distance. SOCS2 is involved in regulating IL-3, IL2-mediated, and Jak-STAT signalling pathways. Signalling cytokines is paramount to Th2 response such as IL3 and IL4, which in turn control immunoglobulin E (IgE) production. CD4+ T cells from *Schistosoma mansoni*-infected SOCS2^-/-^ mice expressed high Type-2 responses after challenge: high levels of IgE, Type-2 responses, eosinophilia and inflammatory pathology compared to wild-type individuals [67]. This gene has also been reported as being differentially expressed in abomasal lymph node (ALN) of resistant Scottish Blackface lambs infected with *Teladorsagia circumcincta* compared to controls [68].

UBE2N has even wider implications, and it has been associated to 14 biological pathways (S3 Table), including eight signalling pathways, three toll-like receptor cascade and two Class I MHC mediated antigen processing and presentation, and FCεRI (IgE high-affinity receptor) mediated NF-kB (nuclear factor kappa-light-chain-enhancer of activated B cells) activation. Ubiquitination regulates many biological processes, immune response being no exception, by causing post-translational modifications and it has been suggested that pathogens take advantage of the ubiquitin pathway in order to circumvent host immune system [69].

OAR3_134032158 showed to be located at 102,539 bp distance from the GALNT4 gene, UDPNacetylalphaD galactosamine:polypeptide N acetylgalactosaminyltransferase 4 (GalNAcT4) involved in mucin-type O, and ATP2B1 is positioned at 184,292bp distance from OAR3_132008863 (Table 3). Plasma membrane 1 (ATP2B1) is involved in haemostasis and platelet haemostasis pathways. OAR1_28678534 was found at 621,028 bp of LRP8, low-density lipoprotein receptor related protein 8, apolipoprotein e receptor, which has been implicated in haemostasis and platelet haemostasis pathways. This gene has been shown to affect clot formation in knockout mice *in vivo* studies [70]. The ATP2B1 and LRP8 findings are of importance as a halt on bleeding could severely impair the constant blood supply from hosts to adult *H*. *contortus*, hindering parasite feeding and survival.

OAR1_25121292 is at 656,565 bp distance from the epidermal growth factor receptor pathway substrate 15 (EPS15) gene, involved in cell secretion and endocytosis, and also found to be highly expressed in mesenteric lymph nodes of resistant cattle to mixed infections of *Ostertagia/Cooperia/Nematodirus* [71].

More importantly, all genes from Table 3 were expressed in lamb and ewe abomasa, mesenteric lymph nodes (MLN), and Peyer's patch, tissues involved in the immune response against parasite infections. Interestingly, MUC15 was the only gene not to be expressed in lamb abomasum. Lambs are born naïve and develop their immune system by continuous exposure to parasites while grazing. By comparing tissue expression profiles from the adult and lamb derived BAM files, it is possible there could be age-related differences in gene expression. If MUC15 is indeed not expressed in lamb abomasum, as it is in adult sheep with developed immune system, then our preliminary observation from this limited data set might suggest the lack of MUC15 expression is a potential mechanism that facilitates a successful parasite infection usually seen in lambs. However, there is no previous information about the animals (age, portion of tissue used for analyses) used to generate the data in the BAM files, and it is possible the lack of MUC15 expression in lamb abomasum could be due to an artefact of a particular individual.

### Suggested response mechanism

At this moment it can only be hypothesised that genes involved in immune cell signalling (such as SOCS2, UBE2N, and EPS15) could favour Th2 cytokine production to increase effector cells (eosinophilia and mastocytosis) and humoral response (high IgE levels) at the site of infection so individuals could become more resistant to gastrointestinal parasite infections. The identification of genes involved in mucin biosynthesis and haemostasis pathways further suggests genetic variants affecting these immunological pathways might also help to establish host resistance. Haemostasis, either induced by the immune response build up or by genes like ATP2B1 and LRP8, could stop bleeding, deterring parasite feeding, or even helping to maintain packed cell volume levels, important for host recovery. Additionally, increasing mucus production by the action of genes like MUC15 and GALNT4, could accelerate parasite expulsion. These findings might serve as side information for areas such as vaccine or immunomodulatory product development.

Taken these pathways together they could help to control parasite burden in more resistant Red Maasai x Dorper individuals. The true association between relevant SNP markers and the genes described previously lies on linkage disequilibrium, which is yet to be tested.

The results suggest other genomic regions besides those within the MHC genes and near the interferon gamma gene are associated to host parasite resistance (S4 Table). Sheep adapted to tropical environments, exposed to high temperatures and humidity, low availability of good-quality pasture, and high parasite transmission levels, might have adapted to parasite infections by developing alternative strategies that guarantee survival. Genetic associations to FEC do exist, however, it seems unlikely to find a gastrointestinal resistance marker that would serve all sheep breeds, because of differences in allele frequencies and in linkage disequilibrium. Our data suggest that variation in SNP markers closely located to a number of important immune cell signalling, mucus production and haemostasis pathways are the main contributors to phenotypic differences in parasite resistance in Red Maasai x Dorper population.

## Conclusions

Several SNP markers have been shown to be associated with packed cell volume, live weight and faecal egg counts. Top markers explained 2.17%, 3.7% and 2.33% of the phenotypic variation for AVPCV, AVLWT and AVFEC, respectively, and association to AVFEC did not cause significant differences on AVPCV nor AVLWT.

Our results also indicate that important genes known to be involved in parasite immune response were located close to the most significant SNP markers in this resource population. These findings suggest that genetic variation in multiple genes involved in the three important immune response pathways of cytokine signalling, haemostasis and mucus biosynthesis probably determine the host response to parasite infection.

## Supporting Information

S1 Fig Manhattan plot for AVFEC using EMMAX additive model genome-wide association analyses (permutation results in grey) and their BLUPf90/PostGSf90 equivalent (permutation results in grey), showing significant markers on sheep chromosomes (OAR) 01, 02, 03, 06, 11, 12, and 15 (a-g). Dashed red line corresponds to the SNP with the highest −log10 p-value.
(PDF)

S1 Table Distribution of SNP markers after Genome Studio and PLINK filtering. (DOC)

S2 Table List of GenBank accession number and names of genes of the RefSeq sequences located within a ±1Mbp distance from significant SNP markers. (PDF)

S3 Table List of genes located close to significant SNP markers and which biological pathways they belong to. (PDF)

S4 Table List of references of previous studies (QTL, candidate gene or GWAS) on genetic traits related to sheep resistance to gastrointestinal parasite infections. (DOC)
