# Supplementary material for: Identification of Novel Loci Associated with Gastrointestinal Parasite Resistance in a Red Maasai x Dorper Backcross Population
Source: PLoS One. 2015 Apr 13;10(4):e0122797. doi: 10.1371/journal.pone.0122797 (PMC4395112; doi:10.1371/journal.pone.0122797)
Supplement: S1 Fig — Dashed red line corresponds to the SNP with the highest −log10 p-value. (PDF) [file pone.0122797.s001.pdf]

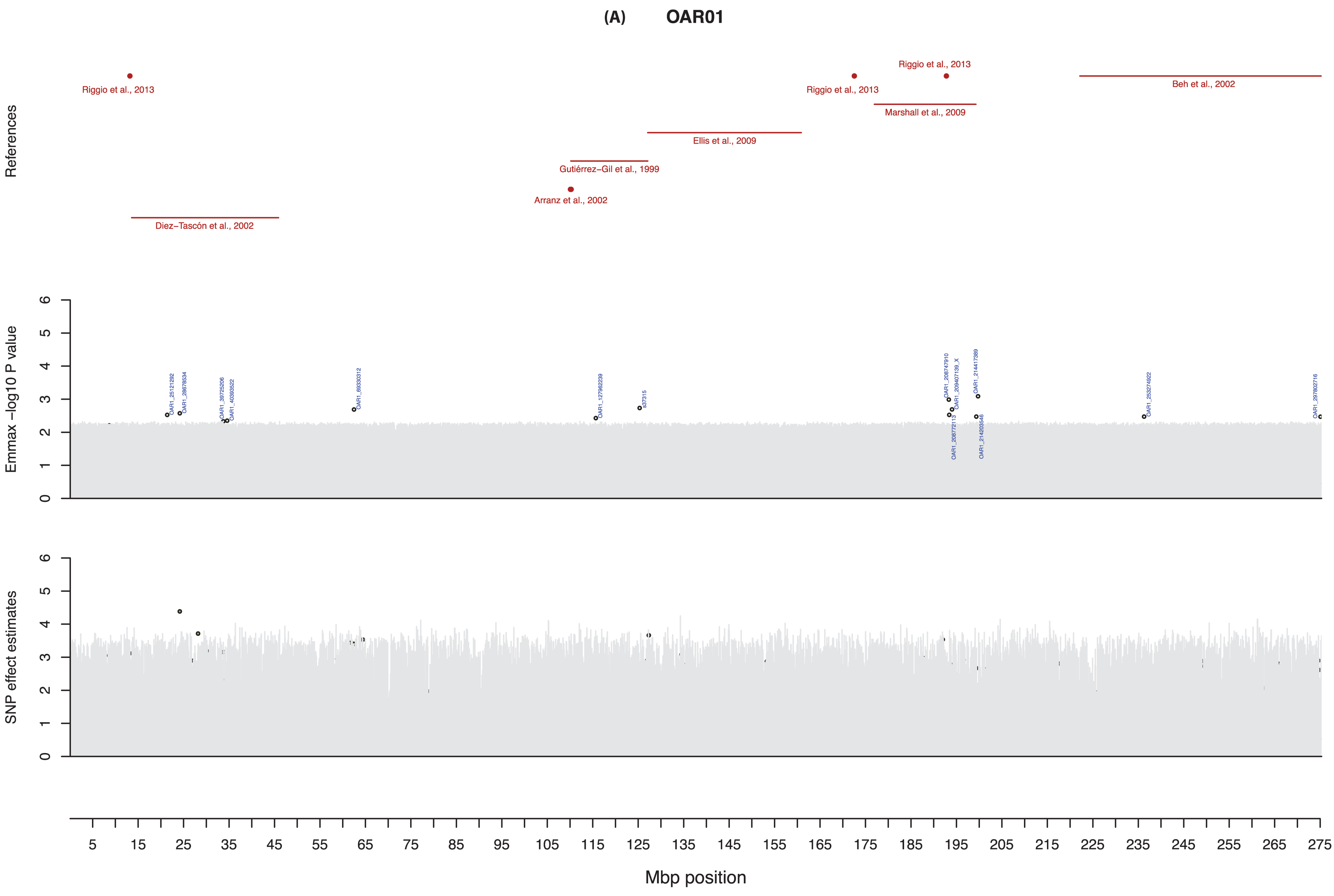

(A) OAR01
References
Riggio et al., 2013
Diez–Tascón et al., 2002
Arranz et al., 2002
Gutiérrez–Gil et al., 1999
Ellis et al., 2009
Riggio et al., 2013
Riggio et al., 2013
Marshall et al., 2009
Beh et al., 2002
Emmax −log10 P value
OAR1_25121292
OAR1_28678534
OAR1_39725206
OAR1_40393522
OAR1_69330312
OAR1_127962239
s37315
OAR1_208747910
OAR1_208772113
OAR1_209407139_X
OAR1_214417389
OAR1_214203546
OAR1_253274922
OAR1_297802716
SNP effect estimates
Mbp position

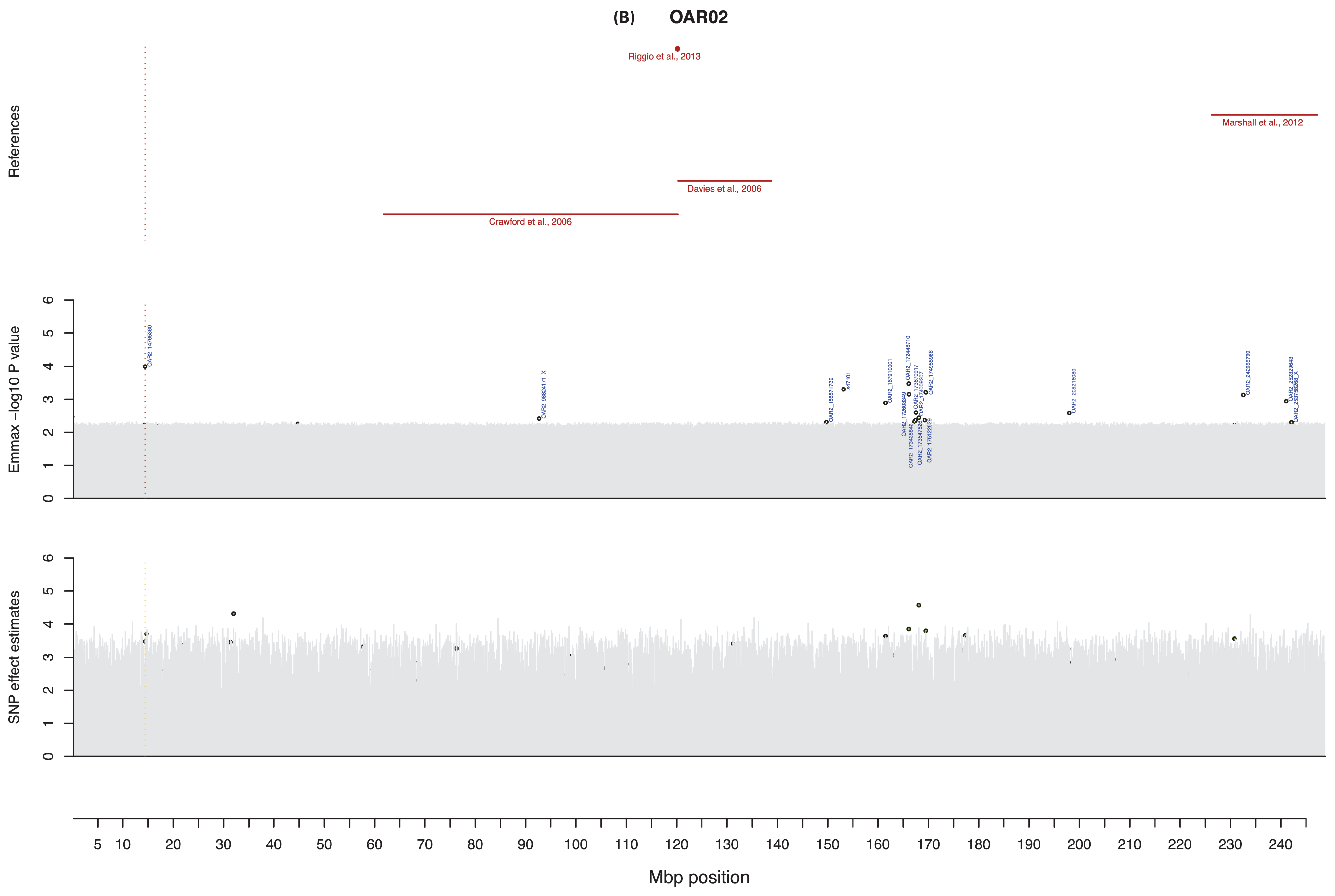

(B) OAR02
References
Riggio et al., 2013
Marshall et al., 2012
Davies et al., 2006
Crawford et al., 2006
Emmax −log10 P value
OAR2_14765360
OAR2_98824171_X
OAR2_156571739
s47101
OAR2_167910001
OAR2_172448710
OAR2_172603349
OAR2_173435842
OAR2_173670917
OAR2_173547626
OAR2_174009207
OAR2_174955986
OAR2_175122529
OAR2_205216089
OAR2_242055799
OAR2_252329643
OAR2_253758268_X
SNP effect estimates
Mbp position

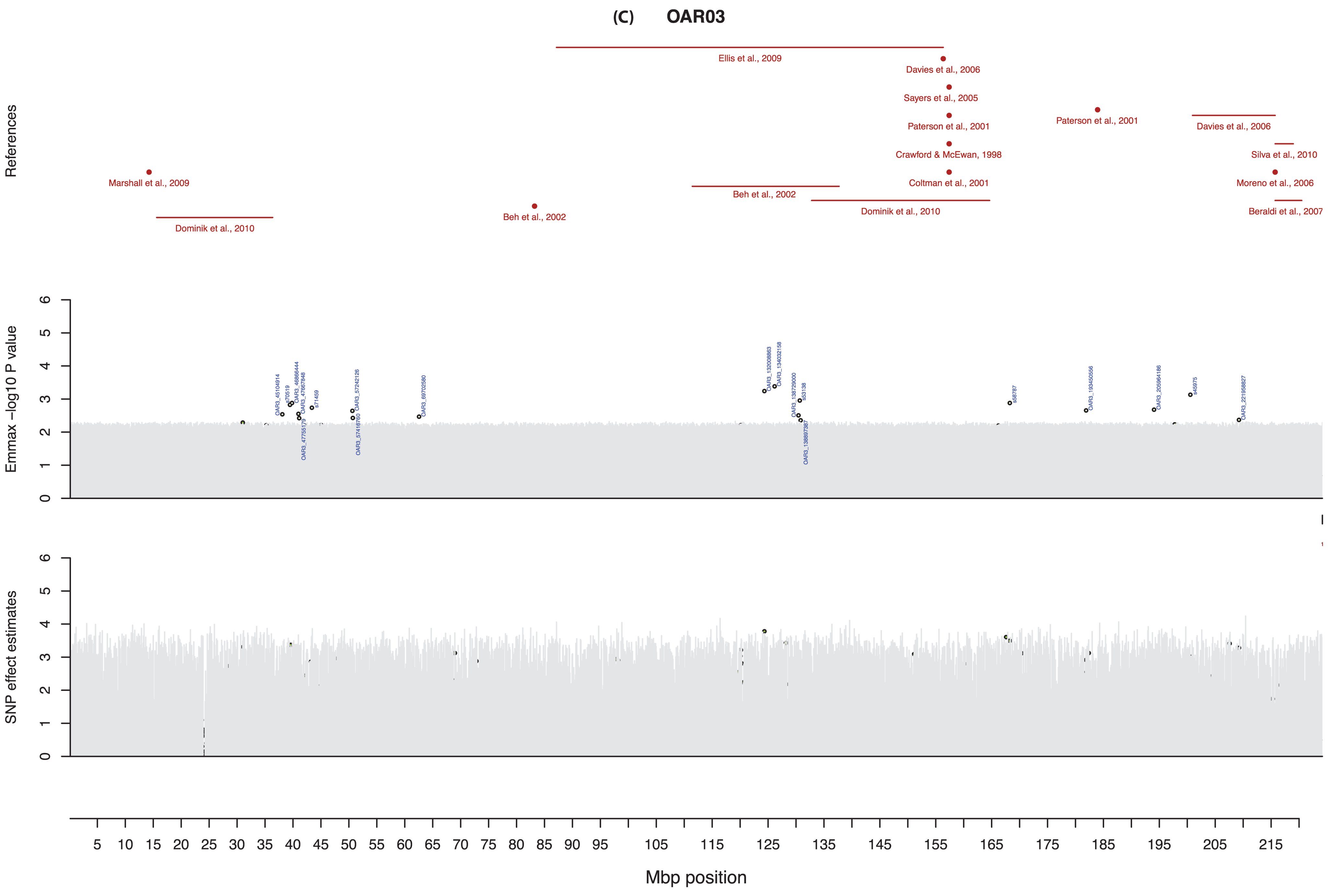

(C) OAR03
References
Ellis et al., 2009
Davies et al., 2006
Sayers et al., 2005
Paterson et al., 2001
Crawford & McEwan, 1998
Coltman et al., 2001
Paterson et al., 2001
Davies et al., 2006
Silva et al., 2010
Moreno et al., 2006
Beraldi et al., 2007
Marshall et al., 2009
Dominik et al., 2010
Beh et al., 2002
Beh et al., 2002
Dominik et al., 2010
Emmax −log10 P value
OAR3_45104914
s70519
OAR3_46866444
OAR3_47667848
OAR3_47755179
s71459
OAR3_57242126
OAR3_57416760
OAR3_69702580
OAR3_132008863
OAR3_134032158
OAR3_138729000
s53138
OAR3_138897387
s58787
OAR3_193450556
OAR3_205964186
s45975
OAR3_221958827
SNP effect estimates
Mbp position

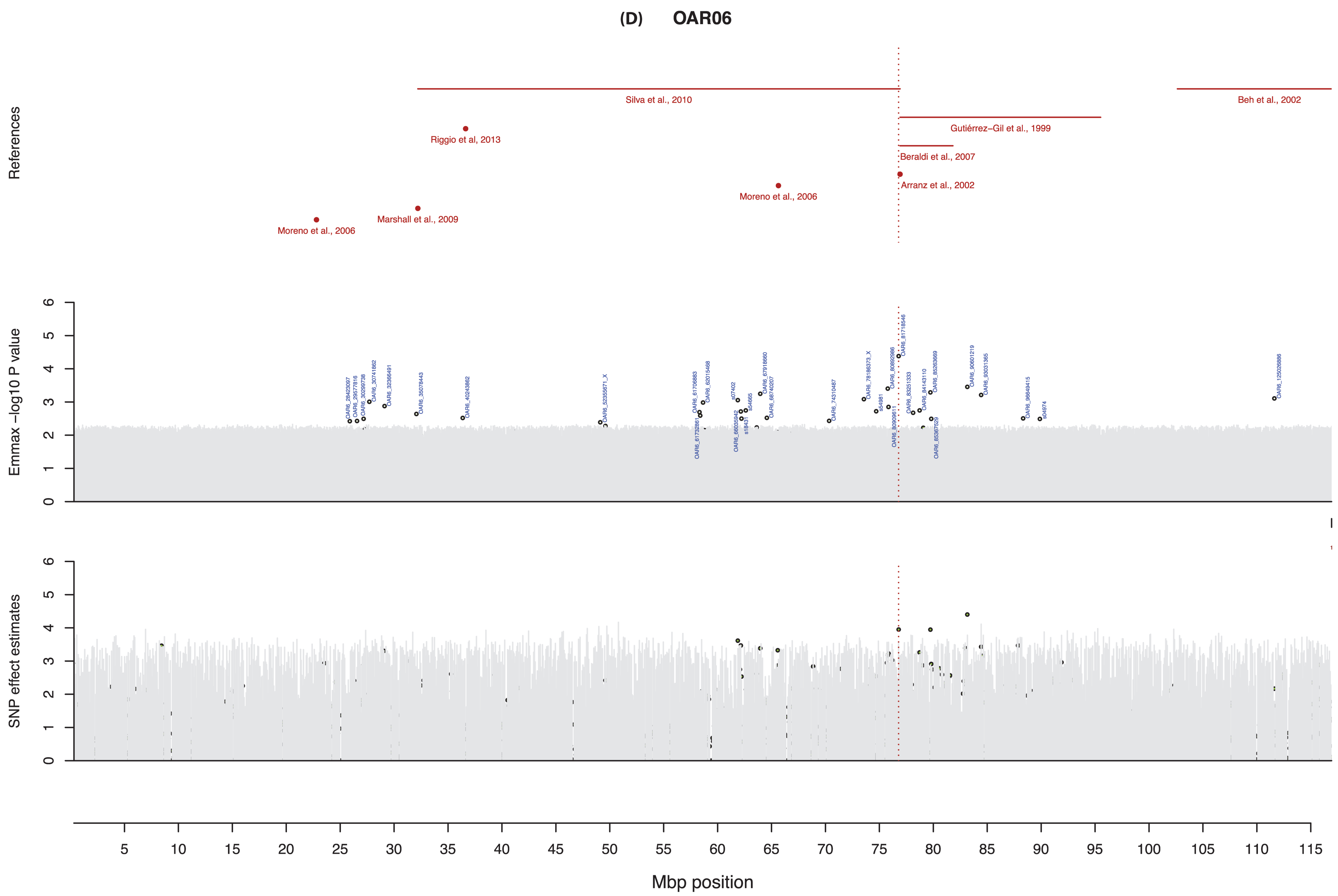

(D) OAR06
References
Silva et al., 2010
Beh et al., 2002
Riggio et al, 2013
Gutiérrez–Gil et al., 1999
Beraldi et al., 2007
Arranz et al., 2002
Moreno et al., 2006
Marshall et al., 2009
Moreno et al., 2006
Emmax −log10 P value
SNP effect estimates
Mbp position
OAR6_28423097
OAR6_29577816
OAR6_30299738
OAR6_30741862
OAR6_32366491
OAR6_35078443
OAR6_40243862
OAR6_52355671_X
OAR6_61706883
OAR6_61732861
OAR6_62015468
OAR6_66035942
s07402
s18431
s54665
OAR6_67918660
OAR6_68740207
OAR6_74310487
OAR6_78186373_X
s54981
OAR6_80909611
OAR6_80892986
OAR6_81718546
OAR6_83251333
OAR6_84143110
OAR6_85263669
OAR6_85367529
OAR6_90601219
OAR6_93031365
OAR6_96649415
s04974
OAR6_125026886

(E) OAR11

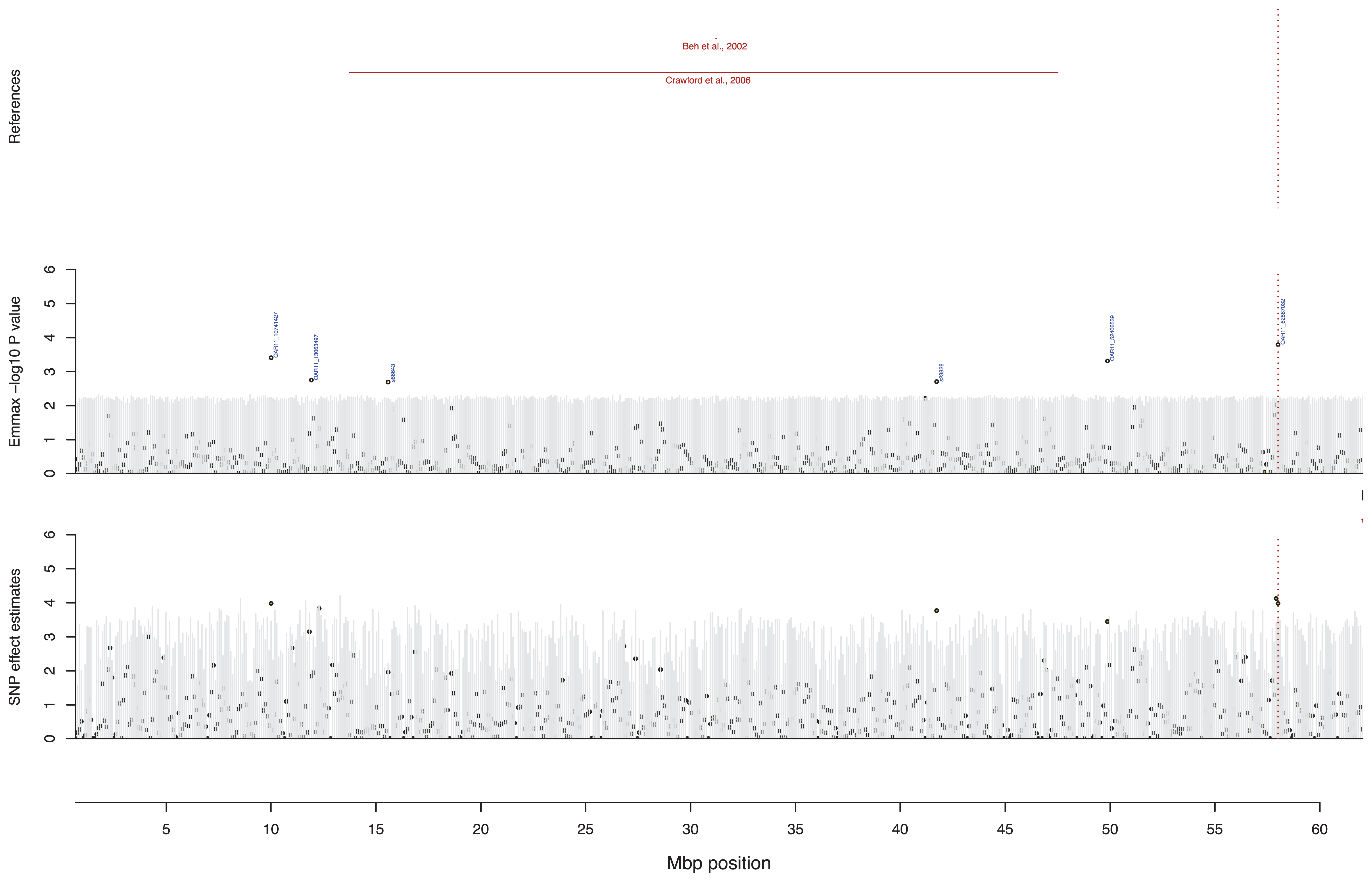

References
Beh et al., 2002
Crawford et al., 2006
Emmax −log10 P value
OAR11_10741427
OAR11_13063497
s66643
s23828
OAR11_52406539
OAR11_62887032
SNP effect estimates
Mbp position

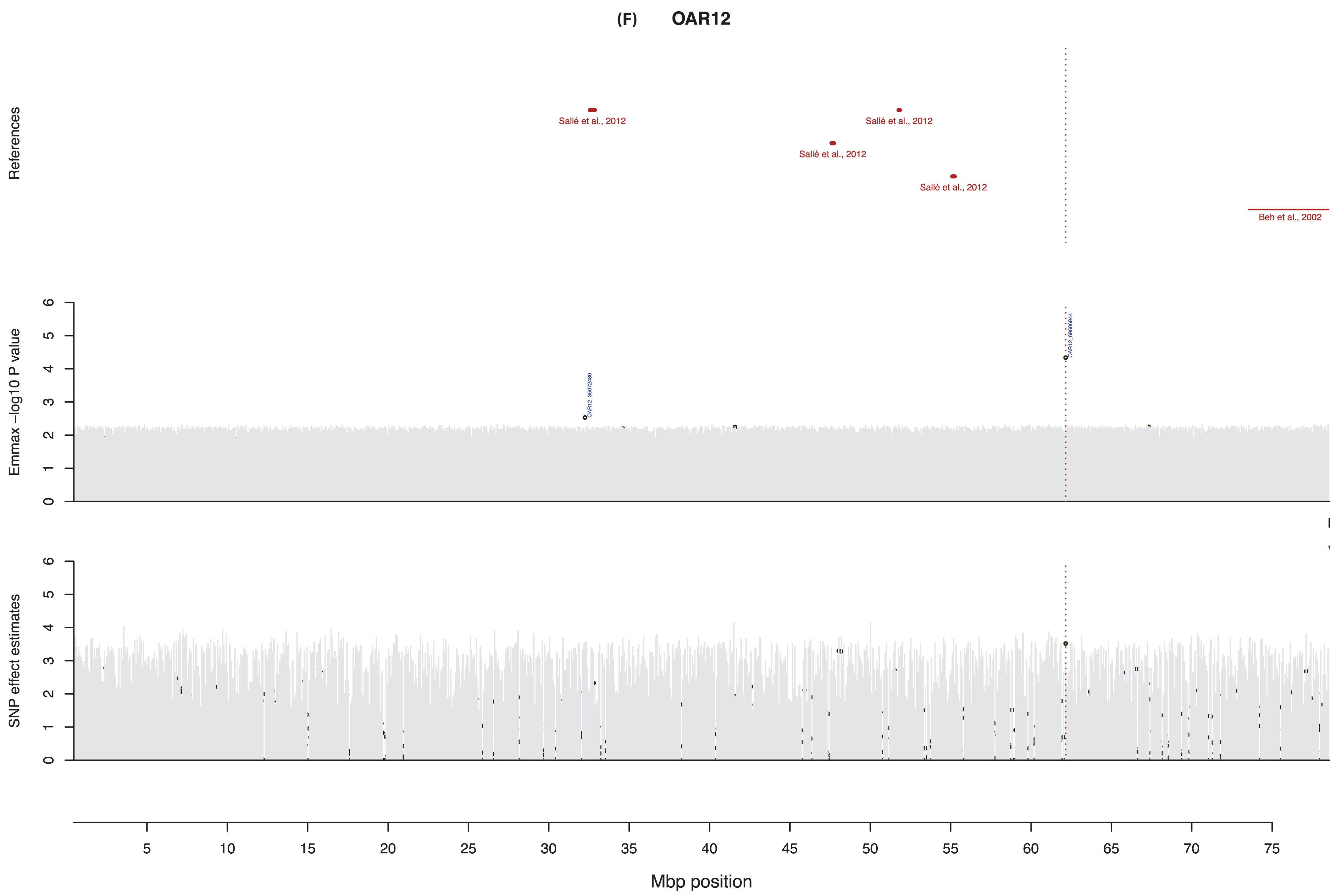
(F) OAR12
References
Sallé et al., 2012
Sallé et al., 2012
Sallé et al., 2012
Sallé et al., 2012
Beh et al., 2002
Emmax −log10 P value
OAR12_35972480
OAR12_69606944
SNP effect estimates
Mbp position

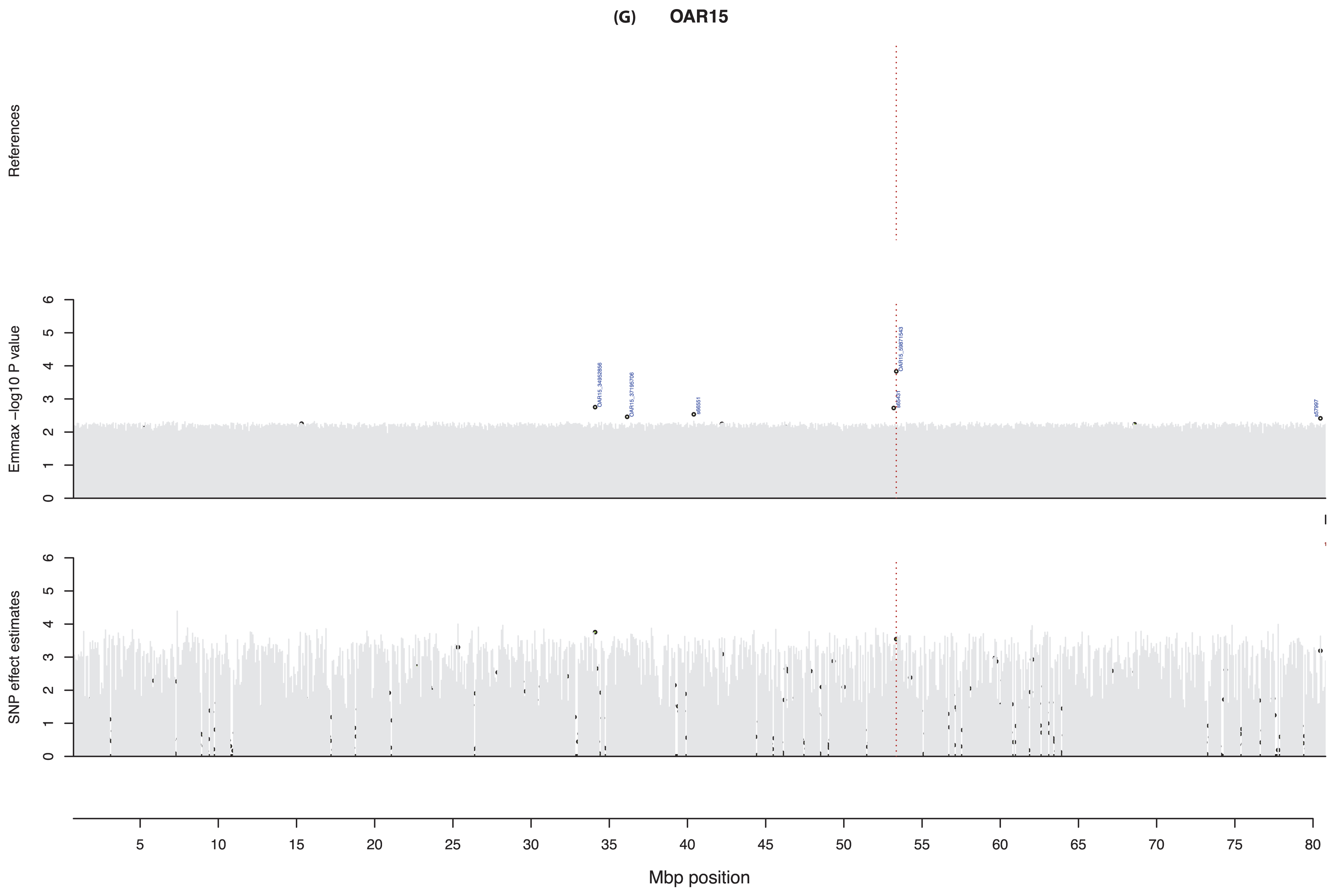
(G) OAR15
References
Emmax −log10 P value
SNP effect estimates
OAR15_34952856
OAR15_37195706
s66551
OAR15_59871543
s65431
s57997
Mbp position
0
1
2
3
4
5
6
5
10
15
20
25
30
35
40
45
50
55
60
65
70
75
80
