# Supplementary material for: Identification of Novel Loci Associated with Gastrointestinal Parasite Resistance in a Red Maasai x Dorper Backcross Population
Source: PLoS One. 2015 Apr 13;10(4):e0122797. doi: 10.1371/journal.pone.0122797 (PMC4395112; doi:10.1371/journal.pone.0122797)
Supplement: S1 Table — (DOC) [file pone.0122797.s002.doc]

**S1 Table. Distribution of SNP markers after Genome Studio and PLINK filtering.**

| **OAR** | **Average SNP interval** | **Minimum SNP interval** | **Maximum SNP interval** | **Number of SNP markers** |
| --- | --- | --- | --- | --- |
| **1** | 77,278.57 | 2,385 | 990,577 | 3,563 |
| **2** | 69,764.77 | 1,780 | 778,817 | 3,565 |
| **3** | 75,453.92 | 3,269 | 874,051 | 2,970 |
| **4** | 70,843.76 | 2,701 | 766,361 | 1,681 |
| **5** | 75,302.31 | 65 | 958,197 | 1,417 |
| **6** | 67,137.58 | 2,024 | 918,977 | 1,738 |
| **7** | 70,154.38 | 2,198 | 545,701 | 1,419 |
| **8** | 66,750.89 | 2,615 | 873,178 | 1,354 |
| **9** | 71,818.19 | 3,658 | 766,982 | 1,312 |
| **10** | 77,317.77 | 2,462 | 1,307,897 | 1,090 |
| **11** | 91,438.40 | 5,128 | 562,068 | 672 |
| **12** | 75,905.31 | 1,945 | 981,963 | 1,030 |
| **13** | 82,791.24 | 5,256 | 596,736 | 1,002 |
| **14** | 102,116.08 | 36 | 705,599 | 612 |
| **15** | 81,950.25 | 2,131 | 781,472 | 978 |
| **16** | 80,368.62 | 2,462 | 894,534 | 886 |
| **17** | 89,084.47 | 3,951 | 478,276 | 809 |
| **18** | 79,349.56 | 5,598 | 604,161 | 850 |
| **19** | 85,405.49 | 5,053 | 460,083 | 704 |
| **20** | 77,598.39 | 5,551 | 665,910 | 645 |
| **21** | 103,315.62 | 2,843 | 2,487,816 | 483 |
| **22** | 73,560.30 | 2,106 | 1,106,265 | 687 |
| **23** | 95,874.83 | 6,486 | 744,806 | 647 |
| **24** | 104,565.82 | 6,428 | 660,340 | 395 |
| **25** | 73,590.83 | 5,560 | 1,220,251 | 616 |
| **26** | 78,205.77 | 5,570 | 378,384 | 561 |
| **Total** |  |  |  | 31,686 |
| **Average interval (bp)** | 80,652 |  |  |  |
| **Minimum interval (bp)** | 66,751 |  |  |  |
| **Maximum interval (bp)** | 104,566 |  |  |  |

54,241 SNP markers on OvineSNP50K BeadChip

19,365 Markers failed call rate (Genome Studio) 35.7%

34,876 Markers in PLINK input file

627 Markers failed missingness test (GENO>0.1) 1.16%

917 Markers failed frequency test (MAF<0.01) 1.69%

1,646 Chromosome X SNP markers 3.05%

31,686 Total number of SNP markers after filtering

58.42% of OvineSNP50K BeadChip
