# Supplementary material for: Identification of Novel Loci Associated with Gastrointestinal Parasite Resistance in a Red Maasai x Dorper Backcross Population
Source: PLoS One. 2015 Apr 13;10(4):e0122797. doi: 10.1371/journal.pone.0122797 (PMC4395112; doi:10.1371/journal.pone.0122797)
Supplement: S2 Table — (PDF) [file pone.0122797.s003.pdf]

S2 Table. List of GenBank accession number and names of genes of the RefSeq sequences located within a ±1Mbp distance from significant SNP markers.

OAR/ Cluster	GENBANK Accession #	Gene code	Gene Name	*Homo sapiens*[1]	*Bos taurus*[2]
**01/01**	NM_001144775	ELAV	ELAV (embryonic lethal abnormal vision Drosophila)-like 4 (Hu antigen D)	*	
**01/01**	NM_007051	TNFRSF6	Fas (TNFRSF6) associated factor 1	*	
**01/01**	NM_078626	CDKN2C	cyclin-dependent kinase inhibitor 2C (p18 inhibits CDK4)	*	*
**01/01**	NM_001159969	EPS15	epidermal growth factor receptor pathway substrate 15	*	*
**01/01**	NM_001981	EPS15	epidermal growth factor receptor pathway substrate 15	*	*
**01/01**	NM_148908	OSBPL9	oxysterol binding protein-like 9	*	*
**01/01**	NM_014372	RNF11	ring finger protein 11	*	*
**01/02**	NM_014762	DHCR24	24-dehydrocholesterol reductase	*	*
**01/02**	NM_033067	DMRTB1	DMRT-like family B with proline-rich C-terminal 1	*	
**01/02**	NM_015547	ACOT11	acyl-CoA thioesterase 11	*	*
**01/02**	NM_001110533	C1orf177	chromosome 1 open reading frame 177	*	
**01/02**	NM_001039716	DIO1	deiodinase iodothyronine type I	*	*
**01/02**	NM_176782	FAM151A	family with sequence similarity 151 member A	*	
**01/02**	NM_016126	HSPB11	heat shock protein family B (small) member 11	*	*
**01/02**	NR_002314	FLJ40434	hypothetical FLJ40434	*	
**01/02**	NM_017522	LRP8	low density lipoprotein receptor-related protein 8 apolipoprotein e receptor	*	*
**01/02**	NM_033300	LRP8	low density lipoprotein receptor-related protein 8 apolipoprotein e receptor	*	*
**01/02**	NM_153703	PODN	podocan	*	*
**01/02**	NM_152268	PARS2	prolyl-tRNA synthetase 2 mitochondrial (putative)	*	
**01/02**	NM_006671	SLC1A7	solute carrier family 1 (glutamate transporter) member 7	*	*
**01/02**	NM_001007099	SCP2	sterol carrier protein 2	*	*
**01/02**	NM_004872	TMEM59	transmembrane protein 59	*	*
**01/02**	NM_015306	USP24	ubiquitin specific peptidase 24	*	
**01/03**	NM_198505	ATP13A5	ATPase type 13A5	*	
**01/03**	NM_178335	CCDC50	coiled-coil domain containing 50	*	*
**02/01**	NM_004235	KLF4	Kruppel-like factor 4 (gut)	*	*
**02/01**	NM_003640	IKBKAP	inhibitor of kappa light polypeptide gene enhancer in B-cells kinase complex-associated protein	*	
**02/02**	NM_024659	GTDC1	glycosyltransferase-like domain containing 1	*	*
**02/03**	NM_001142615	EHBP1	EH domain binding protein 1	*	
**02/03**	NM_001002243	AFTPH	aftiphilin	*	
**02/03**	NM_017657	AFTPH	aftiphilin	*	
**02/03**	NM_001005739	VPS54	vacuolar protein sorting 54 homolog (S. cerevisiae)	*	*
**03/02**	NM_001001323	ATP2B1	ATPase Ca++ transporting plasma membrane 1	*	*

**03/02**	NM_003774	GALNT4	UDP-N-acetyl-alpha-D-galactosamine:polypeptide N-acetylgalactosaminyltransferase 4 (GalNAc-T4)	*	*
**03/02**	NM_172240	WDR51B	WD repeat domain 51B	*	
**03/02**	NM_001009894	C12orf29	chromosome 12 open reading frame 29	*	
**03/02**	NM_181783	TMTC3	transmembrane and tetratricopeptide repeat containing 3	*	
**03/03**	NM_003877	SOCS2	suppressor of cytokine signaling 2	*	*
**03/03**	NM_020698	TMCC3	transmembrane and coiled-coil domain family 3	*	*
**03/03**	NM_003348	UBE2N	ubiquitin-conjugating enzyme E2N (UBC13 homolog yeast)	*	*
**06**	NM_006095	APLT	ATPase aminophospholipid transporter (APLT) class I type 8A member 1	*	
**06**	NM_015115	DCUN1D4	DCN1 defective in cullin neddylation 1 domain containing 4 (S. cerevisiae)	*	*
**06**	NM_133267	GSX2	GS homeobox 2	*	*
**06**	NM_001144990	KIAA1239	KIAA1239	*	
**06**	NM_001112720	LIMCH1	LIM and calponin homology domains 1	*	*
**06**	NM_018177	N4BP2	NEDD4 binding protein 2	*	
**06**	NM_024677	NSUN7	NOL1/NOP2/Sun domain family member 7	*	
**06**	NM_001168254	OCIAD1	OCIA domain containing 1	*	*
**06**	NM_001079842	OCIAD1	OCIA domain containing 1	*	*
**06**	NM_001014446	OCIAD2	OCIA domain containing 2	*	*
**06**	NM_001100400	PDS5A	PDS5 regulator of cohesion maintenance homolog A (S. cerevisiae)	*	
**06**	NM_023940	RASL11B	RAS-like family 11 member B	*	*
**06**	NM_181806	AASDH	aminoadipate-semialdehyde dehydrogenase	*	
**06**	NM_001166053	APBB2	amyloid beta (A4) precursor protein-binding family B member 2	*	*
**06**	NM_001166052	APBB2	amyloid beta (A4) precursor protein-binding family B member 2	*	*
**06**	NM_001166050	APBB2	amyloid beta (A4) precursor protein-binding family B member 2	*	*
**06**	NM_001166051	APBB2	amyloid beta (A4) precursor protein-binding family B member 2	*	*
**06**	NM_025009	CEP135	centrosomal protein 135kDa	*	*
**06**	NM_032313	C4orf14	chromosome 4 open reading frame 14	*	
**06**	NM_174921	C4orf34	chromosome 4 open reading frame 34	*	
**06**	NM_004898	CLOCK	clock homolog (mouse)	*	
**06**	NM_012110	CHIC2	cysteine-rich hydrophobic domain 2	*	*
**06**	NM_130902	COX7B2	cytochrome c oxidase subunit VIIb2	*	*
**06**	NM_006870	DSTN	destrin (actin depolymerizing factor)	*	
**06**	NM_178237	EXOC1	exocyst complex component 1	*	
**06**	NM_138389	FAM114A1	family with sequence similarity 114 member A1	*	
**06**	NM_000807	GABRA2	gamma-aminobutyric acid (GABA) A receptor alpha 2	*	*
**06**	NM_173536	GABRG1	gamma-aminobutyric acid (GABA) A receptor gamma 1	*	*
**06**	NM_025087	FLJ21511	hypothetical protein FLJ21511	*	
**06**	NM_015990	KLHL5	kelch-like 5 (Drosophila)	*	
**06**	NM_015236	LPHN3	latrophilin 3	*	*
**06**	NM_032622	LNX1	ligand of numb-protein X 1	*	*
**06**	NM_002703	PPAT	phosphoribosyl pyrophosphate amidotransferase	*	*
**06**	NM_006206	PDGFRA	platelet-derived growth factor receptor alpha polypeptide	*	*
**06**	NM_002913	RFC1	replication factor C (activator 1) 1 145kDa	*	
**06**	NM_001017	RPS13	ribosomal protein S13 pseudogene 8 ribosomal protein S13 ribosomal protein S13 pseudogene 2	*	
**06**	NM_000232	SGCB	sarcoglycan beta (43kDa dystrophin-associated glycoprotein)	*	*

**06**	NM_001093772	KIT	similar to Mast/stem cell growth factor receptor precursor (SCFR) (Proto-oncogene tyrosine-protein kinase Kit) (c-kit) (CD117 antigen); v-kit Hardy-Zuckerman 4 feline sarcoma viral oncogene homolog	*	*
**06**	NM_152401	PDCL2	similar to hCG2040277; phosducin-like 2	*	*
**06**	NM_030956	TLR10	toll-like receptor 10	*	*
**06**	NM_001017388	TLR10	toll-like receptor 10	*	*
**06**	NM_024943	TMEM156	transmembrane protein 156	*	*
**06**	NM_004181	UCHL1	ubiquitin carboxyl-terminal esterase L1 (ubiquitin thiolesterase)	*	*
**06**	NM_175619	ZAR1	zygote arrest 1	*	*
**12**	NM_201569	SMG7	Smg-7 homolog nonsense mediated mRNA decay factor (C. elegans)	*	*
**12**	NM_203454	APOBEC4	apolipoprotein B mRNA editing enzyme catalytic polypeptide-like 4 (putative)	*	
**12**	NM_030933	SHCBP1L	chromosome 1 open reading frame 14	*	
**12**	NM_030806	C1orf21	chromosome 1 open reading frame 21	*	
**12**	NM_002293	LAMC1	laminin gamma 1 (formerly LAMB2)	*	*
**12**	NR_023349	TSEN15	tRNA splicing endonuclease 15 homolog (S. cerevisiae)	*	*
**12**	NM_001127394	TSEN15	tRNA splicing endonuclease 15 homolog (S. cerevisiae)	*	*
**15**	NM_003369	UVRAG	UV radiation resistance associated gene	*	*
**15**	NM_001135091	MUC15	mucin 15 cell surface associated	*	*
**15**	NM_145650	MUC15	mucin 15 cell surface associated	*	*

[1] Database for Annotation, Visualization and Integrated Discovery (DAVID) v6.7 http://david.abcc.ncifcrf.gov/)
[2] *Bos taurus* Oct. 2011 (Baylor Btau_4.6.1/bosTau7) Assembly (http://genome.ucsc.edu/cgi-bin/hgGateway)
