# Supplementary material for: Identification of Novel Loci Associated with Gastrointestinal Parasite Resistance in a Red Maasai x Dorper Backcross Population
Source: PLoS One. 2015 Apr 13;10(4):e0122797. doi: 10.1371/journal.pone.0122797 (PMC4395112; doi:10.1371/journal.pone.0122797)
Supplement: S3 Table — (PDF) [file pone.0122797.s004.pdf]

## S3 Table. List of genes located close to significant SNPs and which biological pathways they belong to.

| BioSystem name | Pathway | UBE2N | SOCS2 | LAMC1 | EPS15 | ATP2B1 | LRP8 | GALNT4 | MUC15 |
|---|---|---|---|---|---|---|---|---|---|
| **Class I MHC mediated antigen processing & presentation** | Antigen processing and presentation | √ | | | | | | | |
| **Haemostasis** | Haemostasis | | | | | √ | √ | | |
| **Platelet haemostasis** | Haemostasis | | | | | √ | √ | | |
| **Focal adhesion** | Laminin signalling | | | √ | | | | | |
| **Laminin interactions** | Laminin signalling | | | √ | | | | | |
| **Mucin type O-Glycan biosynthesis** | Mucin biosynthesis | | | | | | | √ | |
| **O-glycan biosynthesis, mucin type core** | Mucin biosynthesis | | | | | | | √ | |
| **O-linked glycosylation of mucins** | Mucin biosynthesis | | | | | | | √ | √ |
| **Ubiquitin mediated proteolysis** | Proteolysis | √ | | | | | | | |
| **Cytokine Signalling in Immune system** | Cell signalling | √ | √ | | | | | | |
| **IL-3 Signalling Pathway** | Cell signalling | | √ | | | | | | |
| **IL1-mediated signalling events** | Cell signalling | √ | | | | | | | |
| **IL2-mediated signalling events** | Cell signalling | | √ | | | | | | |
| **Interferon Signalling** | Cell signalling | √ | | | | | | | |
| **Interleukin-1 signalling** | Cell signalling | √ | | | | | | | |
| **JAK-STAT signalling pathway** | Cell signalling | | √ | | | | | | |
| **NOD1/2 Signalling Pathway** | Cell signalling | √ | | | | | | | |
| **Signalling by Interleukins** | Cell signalling | √ | | | | | | | |
| **TCR signalling** | Cell signalling | √ | | | | | | | |
| **TNF-alpha/NF-kB Signalling Pathway** | Cell signalling | | | | √ | | | | |
| **TRAF6 mediated IRF7 activation in TLR7/8 or 9 signalling** | Cell signalling | √ | | | | | | | |
| **Endocytosis** | Cell signalling/activation | | | | √ | | | | |
| **FCERI mediated NF-kB activation** | Cell signalling/activation | √ | | | | | | | |
| **Toll Like Receptor 10 (TLR10) Cascade** | Toll-like receptor | √ | | | | | | | |
| **Toll Like Receptor 7/8 (TLR7/8) Cascade** | Toll-like receptor | √ | | | | | | | |
| **Toll-Like Receptors Cascades** | Toll-like receptor | √ | | | | | | | |
| | Total | 14 | 4 | 2 | 2 | 2 | 2 | 3 | 1 |

FLink tool (http://www.ncbi.nlm.nih.gov/Structure/biosystems/docs/biosystems_ about.html)
