# Supplementary material for: Identification of Novel Loci Associated with Gastrointestinal Parasite Resistance in a Red Maasai x Dorper Backcross Population
Source: PLoS One. 2015 Apr 13;10(4):e0122797. doi: 10.1371/journal.pone.0122797 (PMC4395112; doi:10.1371/journal.pone.0122797)
Supplement: S4 Table — (DOC) [file pone.0122797.s005.doc]

**S4 Table. List of references of previous studies (QTL, candidate gene or GWAS) on genetic traits related to sheep resistance to gastrointestinal parasite infections.**

| **OAR** | **Reference** | **Sheep breed** | **Predominant parasite genera** | **Marker intervals/allelic effects and trait** |
| --- | --- | --- | --- | --- |
| **1** | [Arranz et al., 2004](../../../../C:%5CUsers%5Ctads%5CDocuments%5CMy%20Writing%5CManuscripts%5CSheep%20QTL%20for%20parasites%5CBenavides%20sheep%20QTL%5CArranzetal2002.pdf) | Churra | Strongylidae | OAR1 markers at position 153cM and FEC60 |
| **1** | [Arranz et al., 2004](../../../../C:%5CUsers%5Ctads%5CDocuments%5CMy%20Writing%5CManuscripts%5CSheep%20QTL%20for%20parasites%5CBenavides%20sheep%20QTL%5CArranzetal2002.pdf) | Churra | Strongylidae | OAR1 markers at position 37cM (ILSTS044) and IgA |
| **1** | [Beh et al., 2002](../../../../C:%5CUsers%5Ctads%5CDocuments%5CMy%20Writing%5CManuscripts%5CSheep%20QTL%20for%20parasites%5CBenavides%20sheep%20QTL%5Cbehetal2002.pdf) | Peppin Merino | *Trichostrongylus colubriformis* | McM130 - McM357 and FEC2 |
| **1** | [Diez-Tascón et al., 2002](../../../../C:%5CUsers%5Ctads%5CDocuments%5CMy%20Writing%5CManuscripts%5CSheep%20QTL%20for%20parasites%5CBenavides%20sheep%20QTL%5CDiez-Tasconetal2002.pdf) | Romney x Coopworth cross | Trichostrongyle and *Nematodirus battus* | EPCDV010 - ILSTS044 and FEC2 |
| **1** | [Ellis et al., 2009](../../../../C:%5CUsers%5Ctads%5CDocuments%5CMy%20Writing%5CManuscripts%5CSheep%20QTL%20for%20parasites%5CBenavides%20sheep%20QTL%5Cellisetal_proceedings.pdf) | Indonesian Thin Tail x Merino cross | *Haemonchus contortus* | APPO10/BMS574 - MNS94/CSSM4 at 188cM position and FEC |
| **1** | [Gutierrez-Gil et al., 2009](../../../../C:%5CUsers%5Ctads%5CDocuments%5CMy%20Writing%5CManuscripts%5CSheep%20QTL%20for%20parasites%5CBenavides%20sheep%20QTL%5CGutierrez-Giletal2009.pdf) | Spanish Churra | *Teladorsagia circumcincta* | BMS835 - ILSTS044 and IgA |
| **1** | [Gutierrez-Gil et al., 2009](../../../../C:%5CUsers%5Ctads%5CDocuments%5CMy%20Writing%5CManuscripts%5CSheep%20QTL%20for%20parasites%5CBenavides%20sheep%20QTL%5CGutierrez-Giletal2009.pdf) | Spanish Churra | *Teladorsagia circumcincta* | INRA006 - BMS574 and LFEC1 |
| **1** | [Marshall et al., 2009](../../../../C:%5CUsers%5Ctads%5CDocuments%5CMy%20Writing%5CManuscripts%5CSheep%20QTL%20for%20parasites%5CBenavides%20sheep%20QTL%5CMarshalletal2009.pdf) | Merino | *Haemonchus contortus* | DU363924 DK4483A - BMS2572 and MCM37 - MCM137 for FEC |
| **1** | Riggio et al., 2013 | Scottish Blackface | *Teladorsagia circumcincta* | OAR1_15228673 and SFEC16 |
| **1** | Riggio et al., 2013 | Scottish Blackface | *Teladorsagia circumcincta* | OAR1_184805090 and SFEC |
| **1** | Riggio et al., 2013 | Scottish Blackface | *Teladorsagia circumcincta* | OAR1_208295610 and SFEC16 |
| **2** | Crawford et al., 2006 | Romney x Coopworth cross | Trichostrongyle and *Nematodirus* | BM81124 - BMS1341/HH30 and Latrich (sire124) |
| **2** | [Davies et al., 2006](../../../../C:%5CUsers%5Ctads%5CDocuments%5CMy%20Writing%5CManuscripts%5CSheep%20QTL%20for%20parasites%5CBenavides%20sheep%20QTL%5CDaviesetal2006.pdf) | Scottish Blackface | *Teladorsagia circumcincta* | BM81124 - CP79 and *Nematodirus* FEC (September) |
| **2** | Riggio et al., 2013 | Scottish Blackface | *Teladorsagia circumcincta* | OAR2_128324363 and SFEC16 |
| **2** | Marshall et al., 2012 | Red Maasai x Dorper backcross | *Haemonchus contortus* and *Trichostrongylus colubriformis* | INHA - BM2113 and FEC |
| **3** | [Beh et al., 2002](../../../../C:%5CUsers%5Ctads%5CDocuments%5CMy%20Writing%5CManuscripts%5CSheep%20QTL%20for%20parasites%5CBenavides%20sheep%20QTL%5Cbehetal2002.pdf) | Peppin Merinos | *Trichostrongylus colubriformis* | BM827 - OarFCB5 and FEC2 |
| **3** | [Beraldi et al., 2007](../../../../C:%5CUsers%5Ctads%5CDocuments%5CMy%20Writing%5CManuscripts%5CSheep%20QTL%20for%20parasites%5CBenavides%20sheep%20QTL%5Cberaldietal2007.pdf) | Soay | Strongylidae | CSAP39E - CSSME76 and Coccidia in lambs |
| **3** | [Coltman et al., 2001](../../../../C:%5CUsers%5Ctads%5CDocuments%5CMy%20Writing%5CManuscripts%5CSheep%20QTL%20for%20parasites%5CBenavides%20sheep%20QTL%5Ccoltmanetal2001.pdf) | Soay | *Teladorsagia circumcincta* | MS o(IFN)-g*126 allele reduces FEC in lambs, additive effect |
| **3** | [Coltman et al., 2001](../../../../C:%5CUsers%5Ctads%5CDocuments%5CMy%20Writing%5CManuscripts%5CSheep%20QTL%20for%20parasites%5CBenavides%20sheep%20QTL%5Ccoltmanetal2001.pdf) | Soay | *Teladorsagia circumcincta* | MS o(IFN)-g*126 allele increases IgA in lambs, additive effect |
| **3** | Crawford et al., 1997 | Romney | Strongyle and *Nematodirus* | o(IFN)-g*126 allele: FEC reduction |
| **3** | [Davies et al., 2006](../../../../C:%5CUsers%5Ctads%5CDocuments%5CMy%20Writing%5CManuscripts%5CSheep%20QTL%20for%20parasites%5CBenavides%20sheep%20QTL%5CDaviesetal2006.pdf) | Scottish Blackface | *Teladorsagia circumcincta* | KD103 - LYZ and IgA |
| **3** | [Davies et al., 2006](../../../../C:%5CUsers%5Ctads%5CDocuments%5CMy%20Writing%5CManuscripts%5CSheep%20QTL%20for%20parasites%5CBenavides%20sheep%20QTL%5CDaviesetal2006.pdf) | Scottish Blackface | *Teladorsagia circumcincta* | BM6433 - BMS772 and *Nematodirus* FEC (August) |
| **3** | [Davies et al., 2006](../../../../C:%5CUsers%5Ctads%5CDocuments%5CMy%20Writing%5CManuscripts%5CSheep%20QTL%20for%20parasites%5CBenavides%20sheep%20QTL%5CDaviesetal2006.pdf) | Scottish Blackface | *Teladorsagia circumcincta* | CSRD111 - TEXAN15 and Strongyle FEC (October) |
| **3** | [Dominik et al., 2010](../../../../C:%5CUsers%5Ctads%5CDocuments%5CMy%20Writing%5CManuscripts%5CSheep%20QTL%20for%20parasites%5CBenavides%20sheep%20QTL%5CDominiketal2010.pdf) | Romney x Merino backcross | 1st and 2nd *Trichostrongylus colubriformis*, and 3rd *Haemonchus contortus* infection | OarCP34 - CSAP31e and FWEC (first infection) |
| **3** | [Dominik et al., 2010](../../../../C:%5CUsers%5Ctads%5CDocuments%5CMy%20Writing%5CManuscripts%5CSheep%20QTL%20for%20parasites%5CBenavides%20sheep%20QTL%5CDominiketal2010.pdf) | Romney x Merino backcross | 1st and 2nd *Trichostrongylus colubriformis*, and 3rd *Haemonchus contortus* infection | CSAP019 - MAF23 and eosinophil change (secondary infection) |
| **3** | [Ellis et al., 2009](../../../../C:%5CUsers%5Ctads%5CDocuments%5CMy%20Writing%5CManuscripts%5CSheep%20QTL%20for%20parasites%5CBenavides%20sheep%20QTL%5Cellisetal_proceedings.pdf) | Indonesian Thin Tail x Merino cross | *Haemonchus contortus* | BM2818/BMS1953 - UCD52/BL4 and FEC |
| **3** | [Marshall et al., 2009](../../../../C:%5CUsers%5Ctads%5CDocuments%5CMy%20Writing%5CManuscripts%5CSheep%20QTL%20for%20parasites%5CBenavides%20sheep%20QTL%5CMarshalletal2009.pdf) | Merino | *Haemonchus contortus* | ILSTS28 - ILSTS45 and FEC |
| **3** | [Moreno et al., 2006](../../../../C:%5CUsers%5Ctads%5CDocuments%5CMy%20Writing%5CManuscripts%5CSheep%20QTL%20for%20parasites%5CBenavides%20sheep%20QTL%5CMorenoetal2006_wcgalp.pdf) | Design 1: Sarda x Lacaune backcross; Design 2: Black Belly x INRA401 backcross | Design 1: *Haemonchus contortus* and *Teladorsagia circumcincta*; Design 2: *Haemonchus contortus* | Suggestive QTL near position 275.0cM and FEC |
| **3** | Paterson et al., 1999 | Romney divergent lines |  | IFNG - BMS1617 and FEC |
| **3** | [Paterson et al., 2001](../../../../C:%5CUsers%5Ctads%5CDocuments%5CMy%20Writing%5CManuscripts%5CSheep%20QTL%20for%20parasites%5CBenavides%20sheep%20QTL%5CPatersonetal2001_proceedings.pdf) | Romney and Perendale | *Teladorsagia circumcincta*, *Haemonchus contortus*, Nematodirus, and *Trichostrongylus colubriformis* | IFNG 5' SNP (TT) and IFNG ins/del (BB) -19.88% of mean %BV for FEC2; IFNG 5' SNP (CT) IFNG ins/del (AB) +8.58% of mean %BV for FEC2 in Romney sheep |
| **3** | Sayers et al., 2005a | Suffolk | Trichostrongyle and Nematodirus battus | No association between IFNG intron 1 and FEC |
| **3** | Sayers et al., 2005a | Texel | Trichostrongyle and *Nematodirus battus* | IFNG intron 1 x FEC: haplotype B (GTTT)_5_ associated with resistance |
| **3** | [Silva et al., 2011](../../../../C:%5CUsers%5Ctads%5CDocuments%5CMy%20Writing%5CManuscripts%5CSheep%20QTL%20for%20parasites%5CBenavides%20sheep%20QTL%5CSilvaetal2010.pdf) | Red Maasai x Dorper backcross | *Haemonchus contortus* and *Trichostrongylus colubriformis* | CSAP39E - BM2830 and AVFEC |
| **4** | [Marshall et al., 2009](../../../../C:%5CUsers%5Ctads%5CDocuments%5CMy%20Writing%5CManuscripts%5CSheep%20QTL%20for%20parasites%5CBenavides%20sheep%20QTL%5CMarshalletal2009.pdf) | Merino | *Haemonchus contortus* | AC113228 - ILSTS62 and CP26 - BMS648 for FEC |
| **4** | Marshall et al., 2012 | Red Maasai x Dorper backcross | *Haemonchus contortus* and *Trichostrongylus colubriformis* | BMS1788 - MCM2 and FEC |
| **5** | [Arranz et al., 2004](../../../../C:%5CUsers%5Ctads%5CDocuments%5CMy%20Writing%5CManuscripts%5CSheep%20QTL%20for%20parasites%5CBenavides%20sheep%20QTL%5CArranzetal2002.pdf) | Churra | Strongylidae | Significant QTL for OAR5 markers and PCV1 |
| **5** | [Benavides et al., 2002](../../../../C:%5CUsers%5Ctads%5CDocuments%5CMy%20Writing%5CManuscripts%5CSheep%20QTL%20for%20parasites%5CBenavides%20sheep%20QTL%5CBenavidesetal2002.pdf) | Corriedale and Polwarth | *Haemonchus contortus* | CSRD2138*A, OarAE129*I, TGLA176*C, and OarAE129*F alleles reduced FEC in Corriedale sheep |
| **5** | [Benavides et al., 2009](../../../../C:%5CUsers%5Ctads%5CDocuments%5CMy%20Writing%5CManuscripts%5CSheep%20QTL%20for%20parasites%5CBenavides%20sheep%20QTL%5CBenavidesetal2009.pdf) | Corriedale and Polwarth | *Haemonchus contortus* | Reduced FEC when IL-4*B allele replaced by either IL-4*A or IL-4*C alleles |
| **5** | Sallé et al., 2012 | Romane × Martinique Black Belly backcross | *Haemonchus contortus* | OAR5_92638062 - OAR5_92975517 and FEC12t |
| **6** | [Arranz et al., 2004](../../../../C:%5CUsers%5Ctads%5CDocuments%5CMy%20Writing%5CManuscripts%5CSheep%20QTL%20for%20parasites%5CBenavides%20sheep%20QTL%5CArranzetal2002.pdf) | Churra | Strongyllidae | Association for FEC60 and OAR6 markers at position 84cM |
| **6** | [Beh et al., 2002](../../../../C:%5CUsers%5Ctads%5CDocuments%5CMy%20Writing%5CManuscripts%5CSheep%20QTL%20for%20parasites%5CBenavides%20sheep%20QTL%5Cbehetal2002.pdf) | Peppin Merino | *Trichostrongylus colubriformis* | McMA22 - McM214 for FEC1 and FEC2 |
| **6** | [Beraldi et al., 2007](../../../../C:%5CUsers%5Ctads%5CDocuments%5CMy%20Writing%5CManuscripts%5CSheep%20QTL%20for%20parasites%5CBenavides%20sheep%20QTL%5Cberaldietal2007.pdf) | Soay | Strongiloidea | BMS360 - McM140 and FEC |
| **6** | [Gutierrez-Gil et al., 2009](../../../../C:%5CUsers%5Ctads%5CDocuments%5CMy%20Writing%5CManuscripts%5CSheep%20QTL%20for%20parasites%5CBenavides%20sheep%20QTL%5CGutierrez-Giletal2009.pdf) | Spanish Churra | *Teladorsagia circumcincta* | BM4621 - CSN3 and LFEC1 (significant for three families) |
| **6** | [Gutierrez-Gil et al., 2009](../../../../C:%5CUsers%5Ctads%5CDocuments%5CMy%20Writing%5CManuscripts%5CSheep%20QTL%20for%20parasites%5CBenavides%20sheep%20QTL%5CGutierrez-Giletal2009.pdf) | Spanish Churra | *Teladorsagia circumcincta* | BM4621 - CSN3 and LFEC0 (significant for one family) |
| **6** | [Marshall et al., 2009](../../../../C:%5CUsers%5Ctads%5CDocuments%5CMy%20Writing%5CManuscripts%5CSheep%20QTL%20for%20parasites%5CBenavides%20sheep%20QTL%5CMarshalletal2009.pdf) | Merino | *Haemonchus contortus* | MCMA14 - JL1A and FEC |
| **6** | [Moreno et al., 2006](../../../../C:%5CUsers%5Ctads%5CDocuments%5CMy%20Writing%5CManuscripts%5CSheep%20QTL%20for%20parasites%5CBenavides%20sheep%20QTL%5CMorenoetal2006_wcgalp.pdf) | Design 1: Sarda x Lacaune backcross; Design 2: Black Belly x INRA401 backcross | Design 1: *Haemonchus contortus* and *Teladorsagia circumcincta*; Design 2: *Haemonchus contortus* | Suggestive QTLs for FEC at OAR6 markers at 18.0 and 73.0 cM positions |
| **6** | [Silva et al., 2011](../../../../C:%5CUsers%5Ctads%5CDocuments%5CMy%20Writing%5CManuscripts%5CSheep%20QTL%20for%20parasites%5CBenavides%20sheep%20QTL%5CSilvaetal2010.pdf) | Red Maasai x Dorper backcross | *Haemonchus contortus* and *Trichostrongylus colubriformis* | BM1329 - BMS360 and AVFEC |
| **6** | Riggio et al., 2013 | Scottish Blackface | *Teladorsagia circumcincta* | OAR6_40496376 and SFEC |
| **6** | Riggio et al., 2013 | Scottish Blackface | *Teladorsagia circumcincta* | OAR6_40496376 and SFEC16 |
| **7** | [Marshall et al., 2009](../../../../C:%5CUsers%5Ctads%5CDocuments%5CMy%20Writing%5CManuscripts%5CSheep%20QTL%20for%20parasites%5CBenavides%20sheep%20QTL%5CMarshalletal2009.pdf) | Merino | *Haemonchus contortus* | CSAP35E - MCM149 and FEC |
| **7** | Sallé et al., 2012 | Romane × Martinique Black Belly backcross | *Haemonchus contortus* | OAR7_36947114 - OAR7_37429300 and FEC12t |
| **8** | [Crawford et al., 2006](../../../../C:%5CUsers%5Ctads%5CDocuments%5CMy%20Writing%5CManuscripts%5CSheep%20QTL%20for%20parasites%5CBenavides%20sheep%20QTL%5Ccrawfordetal2006.pdf) | Romney x Coopworth cross | Trichostrongyle and *Nematodirus* | BM3215 - Telomere/BM4208 for Latrich and Lsitri (sire124) |
| **8** | Riggio et al., 2013 | Scottish Blackface | *Teladorsagia circumcincta* | OAR8_76576205 and SFEC24 |
| **8** | Riggio et al., 2013 | Scottish Blackface | *Teladorsagia circumcincta* | OAR8_78880291 and SFEC24 |
| **9** | [Arranz et al., 2004](../../../../C:%5CUsers%5Ctads%5CDocuments%5CMy%20Writing%5CManuscripts%5CSheep%20QTL%20for%20parasites%5CBenavides%20sheep%20QTL%5CArranzetal2002.pdf) | Churra | Strongylidae | Association between IgA and OAR9 markers at 50cM position |
| **10** | Riggio et al., 2013 | Scottish Blackface | *Teladorsagia circumcincta* | s52721 and SFEC24 |
| **10** | Riggio et al., 2013 | Scottish Blackface | *Teladorsagia circumcincta* | s56461 and SFEC20 |
| **10** | Riggio et al., 2013 | Scottish Blackface | *Teladorsagia circumcincta* | OAR10_57177592 and SFEC20 |
| **10** | Riggio et al., 2013 | Scottish Blackface | *Teladorsagia circumcincta* | OAR10_91011990 and SFEC24 |
| **10** | Marshall et al., 2012 | Red Maasai x Dorper backcross | *Haemonchus contortus* and *Trichostrongylus colubriformis* | BL1022 and FEC |
| **11** | [Beh et al., 2002](../../../../C:%5CUsers%5Ctads%5CDocuments%5CMy%20Writing%5CManuscripts%5CSheep%20QTL%20for%20parasites%5CBenavides%20sheep%20QTL%5Cbehetal2002.pdf) | Peppin Merino | *Trichostrongylus colubriformis* | TGLA51 and FEC1 |
| **11** | [Crawford et al., 2006](../../../../C:%5CUsers%5Ctads%5CDocuments%5CMy%20Writing%5CManuscripts%5CSheep%20QTL%20for%20parasites%5CBenavides%20sheep%20QTL%5Ccrawfordetal2006.pdf) | Romney x Coopworth cross | Trichostrongyle and *Nematodirus* | BM9202 - Centromere/ETH3 for Lsitri (significant for all sires) |
| **12** | [Beh et al., 2002](../../../../C:%5CUsers%5Ctads%5CDocuments%5CMy%20Writing%5CManuscripts%5CSheep%20QTL%20for%20parasites%5CBenavides%20sheep%20QTL%5Cbehetal2002.pdf) | Peppin Merino | *Trichostrongylus colubriformis* | BM719 - HUJ625 and FEC2 |
| **12** | [Moreno et al., 2006](../../../../C:%5CUsers%5Ctads%5CDocuments%5CMy%20Writing%5CManuscripts%5CSheep%20QTL%20for%20parasites%5CBenavides%20sheep%20QTL%5CMorenoetal2006_wcgalp.pdf) | Design 1: Sarda x Lacaune backcross; Design 2: Black Belly x INRA401 backcross | Design 1: *Haemonchus contortus* and *Teladorsagia circumcincta*; Design 2: *Haemonchus contortus* | Significant QTL for FEC and OAR12 markers |
| **12** | Sallé et al., 2012 | Romane × Martinique Black Belly backcross | *Haemonchus contortus* | OAR12_62226914 - s68186 and FEC12t |
| **12** | Sallé et al., 2012 | Romane × Martinique Black Belly backcross | *Haemonchus contortus* | OAR12_51099743 - OAR12_51339516 and FEC34t |
| **12** | Sallé et al., 2012 | Romane × Martinique Black Belly backcross | *Haemonchus contortus* | OAR12_36077499 - s41448 and FECt_a |
| **12** | Sallé et al., 2012 | Romane × Martinique Black Belly backcross | *Haemonchus contortus* | s23035 - OAR12_56589339 and FECt_a |
| **13** | [Moreno et al., 2006](../../../../C:%5CUsers%5Ctads%5CDocuments%5CMy%20Writing%5CManuscripts%5CSheep%20QTL%20for%20parasites%5CBenavides%20sheep%20QTL%5CMorenoetal2006_wcgalp.pdf) | Design 1: Sarda x Lacaune backcross; Design 2: Black Belly x INRA401 backcross | Design 1: *Haemonchus contortus* and *Teladorsagia circumcincta*; Design 2: *Haemonchus contortus* | Suggestive QTL for FEC and OAR13 markers in both flocks |
| **13** | Sallé et al., 2012 | Romane × Martinique Black Belly backcross | *Haemonchus contortus* | s05259 - s09612 and FEC34t |
| **13** | Sallé et al., 2012 | Romane × Martinique Black Belly backcross | *Haemonchus contortus* | s05603 - s43133 and FEC34t |
| **14** | [Arranz et al., 2004](../../../../C:%5CUsers%5Ctads%5CDocuments%5CMy%20Writing%5CManuscripts%5CSheep%20QTL%20for%20parasites%5CBenavides%20sheep%20QTL%5CArranzetal2002.pdf) | Churra | Strongylidae | Association for FEC0 and OAR14 markers located at 1cM |
| **14** | [Davies et al., 2006](../../../../C:%5CUsers%5Ctads%5CDocuments%5CMy%20Writing%5CManuscripts%5CSheep%20QTL%20for%20parasites%5CBenavides%20sheep%20QTL%5CDaviesetal2006.pdf) | Scottish Blackface | *Teladorsagia circumcincta* | BMS833 - ILSTS002 and *Nematodirus* FEC (August) |
| **14** | [Gutierrez-Gil et al., 2009](../../../../C:%5CUsers%5Ctads%5CDocuments%5CMy%20Writing%5CManuscripts%5CSheep%20QTL%20for%20parasites%5CBenavides%20sheep%20QTL%5CGutierrez-Giletal2009.pdf) | Spanish Churra | *Teladorsagia circumcincta* | TGLA357 - CSRD247 and LFEC0 (significant for two families) |
| **14** | [Silva et al., 2011](../../../../C:%5CUsers%5Ctads%5CDocuments%5CMy%20Writing%5CManuscripts%5CSheep%20QTL%20for%20parasites%5CBenavides%20sheep%20QTL%5CSilvaetal2010.pdf) | Red Maasai x Dorper backcross | *Haemonchus contortus* and *Trichostrongylus colubriformis* | BMS2213 - HAUT14 and AVFEC |
| **16** | Sallé et al., 2012 | Romane × Martinique Black Belly backcross | *Haemonchus contortus* | s04660 - OAR16_26684182 and FEC34t |
| **18** | [Clarke et al., 2001](../../../../C:%5CUsers%5Ctads%5CDocuments%5CMy%20Writing%5CManuscripts%5CSheep%20QTL%20for%20parasites%5CBenavides%20sheep%20QTL%5CClarkeetal2001.pdf) | Merino | *Haemonchus contortus* and *Trichostrongylus colubriformis* (two flocks) | Evidence of association between IgE and FEC for *T. colubriformis* resistance but not for *Haemonchus contortus* flock |
| **18** | [Marshall et al., 2009](../../../../C:%5CUsers%5Ctads%5CDocuments%5CMy%20Writing%5CManuscripts%5CSheep%20QTL%20for%20parasites%5CBenavides%20sheep%20QTL%5CMarshalletal2009.pdf) | Merino | *Haemonchus contortus* | TGLA122 - MCMA26 and FEC |
| **18** | Riggio et al., 2013 | Scottish Blackface | *Teladorsagia circumcincta* | OAR18_34929994 and SFEC |
| **19** | [Moreno et al., 2006](../../../../C:%5CUsers%5Ctads%5CDocuments%5CMy%20Writing%5CManuscripts%5CSheep%20QTL%20for%20parasites%5CBenavides%20sheep%20QTL%5CMorenoetal2006_wcgalp.pdf) | Design 1: Sarda x Lacaune backcross; Design 2: Black Belly x INRA401 backcross | Design 1: *Haemonchus contortus* and *Teladorsagia circumcincta*; Design 2: *Haemonchus contortus* | Significant QTL for OAR19 markers and FEC in design 1 backcross |
| **20** | [Arranz et al., 2004](../../../../C:%5CUsers%5Ctads%5CDocuments%5CMy%20Writing%5CManuscripts%5CSheep%20QTL%20for%20parasites%5CBenavides%20sheep%20QTL%5CArranzetal2002.pdf) | Churra | Strongylidae | Association between FEC and OAR20 markers at 34cM position |
| **20** | [Buitkamp et al., 1996](../../../../C:%5CUsers%5Ctads%5CDocuments%5CMy%20Writing%5CManuscripts%5CSheep%20QTL%20for%20parasites%5CBenavides%20sheep%20QTL%5Cbuitkampetal1996.pdf) | Scottish Blackface | *Ostertagia circumcincta* | MHC Class I and DY locus: 8- and 218-fold FEC reduction in 6-month old lambs |
| **20** | [Charon, 2004](../../../../C:%5CUsers%5Ctads%5CDocuments%5CMy%20Writing%5CManuscripts%5CSheep%20QTL%20for%20parasites%5CBenavides%20sheep%20QTL%5Ccharon2004.pdf) | Heatherheaded sheep | NA | DRB1*482 and DRB1*530 alleles associated to resistance to GI nematodes. DRB1*568 related to susceptibility to parasites. DRB1 RFLP alleles associated to resistance to GI nematodes |
| **20** | [Davies et al., 2006](../../../../C:%5CUsers%5Ctads%5CDocuments%5CMy%20Writing%5CManuscripts%5CSheep%20QTL%20for%20parasites%5CBenavides%20sheep%20QTL%5CDaviesetal2006.pdf) | Scottish Blackface | *Teladorsagia circumcincta* | BM1815 - DRB1 and IgA levels |
| **20** | [Davies et al., 2006](../../../../C:%5CUsers%5Ctads%5CDocuments%5CMy%20Writing%5CManuscripts%5CSheep%20QTL%20for%20parasites%5CBenavides%20sheep%20QTL%5CDaviesetal2006.pdf) | Scottish Blackface | *Teladorsagia circumcincta* | DYA - MCMA36 and Strongyle |
| **20** | [Douch & Outteridge, 1989](../../../../C:%5CUsers%5Ctads%5CDocuments%5CMy%20Writing%5CManuscripts%5CSheep%20QTL%20for%20parasites%5CBenavides%20sheep%20QTL%5CDouch+Outteridge1989.pdf) | Romney Marsh | *Trichostrongylus colubriformis* and *Ostertagia circumcincta* | SY antigens (MHC Class I) and FEC: 1a and 1b alleles with low FEC averages |
| **20** | [Douch & Outteridge, 1989](../../../../C:%5CUsers%5Ctads%5CDocuments%5CMy%20Writing%5CManuscripts%5CSheep%20QTL%20for%20parasites%5CBenavides%20sheep%20QTL%5CDouch+Outteridge1989.pdf) | Romney Marsh | *Trichostrongylus colubriformis* | SY antigens (MHC Class I) and FEC: allele 6 with high FEC average |
| **20** | [Janßen et al., 2002](../../../../C:%5CUsers%5Ctads%5CDocuments%5CMy%20Writing%5CManuscripts%5CSheep%20QTL%20for%20parasites%5CBenavides%20sheep%20QTL%5CJan%25C3%259Fenetal2002.pdf) | Rhönschaf | *Haemonchus contortus* | OarCP73 and PCV: allele A increases PCV by 6.78 and 9.20% in s1 and s2 and allele B reduces 18.7% in s2 |
| **20** | [Janßen et al., 2002](../../../../C:%5CUsers%5Ctads%5CDocuments%5CMy%20Writing%5CManuscripts%5CSheep%20QTL%20for%20parasites%5CBenavides%20sheep%20QTL%5CJan%25C3%259Fenetal2002.pdf) | Rhönschaf | *Haemonchus contortus* | DYMS1 (DYA) and IgL: allele C reduces IgL by 15.6% in s2 |
| **20** | [Janßen et al., 2002](../../../../C:%5CUsers%5Ctads%5CDocuments%5CMy%20Writing%5CManuscripts%5CSheep%20QTL%20for%20parasites%5CBenavides%20sheep%20QTL%5CJan%25C3%259Fenetal2002.pdf) | Rhönschaf | *Haemonchus contortus* | BM1815 and FEC: allele C reduces FEC in 5.56% |
| **20** | [Janßen et al., 2004](../../../../C:%5CUsers%5Ctads%5CDocuments%5CMy%20Writing%5CManuscripts%5CSheep%20QTL%20for%20parasites%5CBenavides%20sheep%20QTL%5CJan%25C3%259Fenetal2004.pdf) | German breed Merinoland | *Haemonchus contortus* | OlaDRBps: B-allele significant effect on haematocrit values and E-allele on 8-week post-infection serum IgL-levels |
| **20** | [Keane et al., 2007](../../../../C:%5CUsers%5Ctads%5CDocuments%5CMy%20Writing%5CManuscripts%5CSheep%20QTL%20for%20parasites%5CBenavides%20sheep%20QTL%5Ckeaneetal2007.pdf) | Perendale, Romney, and Coopworth | Teladorsagia, Trichostrongylus, Cooperia | Homozygous null DQA1 allele associated with susceptibility to parasites |
| **20** | [Luffau et al., 1990](../../../../C:%5CUsers%5Ctads%5CDocuments%5CMy%20Writing%5CManuscripts%5CSheep%20QTL%20for%20parasites%5CBenavides%20sheep%20QTL%5CLuffauetal1990.pdf) | Romanov | *Haemonchus contortus* | Association between FEC and OLA antigens (Hb) |
| **20** | [Outteridge et al., 1988](../../../../C:%5CUsers%5Ctads%5CDocuments%5CMy%20Writing%5CManuscripts%5CSheep%20QTL%20for%20parasites%5CBenavides%20sheep%20QTL%5COutteridgeetal1988.pdf) | Merino | *Trichostrongylus colubriformis* | SY antigens (MHC Class I) and FEC: allele 1 halved FEC after vaccination trial |
| **20** | [Paterson et al., 1998](../../../../C:%5CUsers%5Ctads%5CDocuments%5CMy%20Writing%5CManuscripts%5CSheep%20QTL%20for%20parasites%5CBenavides%20sheep%20QTL%5Cpatersonetal1998.pdf) | Soay | *Teladorsagia circumcincta* | OLADRB*257 allele increased FEC by 104 eggs/g; OLADRB*263 allele reduced FEC by 76 eggs/g; OLADRB*267 allele increased FEC by 96 eggs/g in yearlings |
| **20** | Sayers et al., 2005 | Suffolk | *Teladorsagia circumcincta*, *Trichostrongylus colubriformis*, *Nematodirus battus* | Ovar-DRB1*0203 allele decreased FEC in 45% and Ovar-DRB33 in 30%. Ovar-DRB10 allele increased FEC by 25% |
| **20** | Sayers et al., 2005 | Texel | *Teladorsagia circumcincta*, *Trichostrongylus colubriformis*, *Nematodirus* | No evidence of Ovar-DRB1 association with FEC |
| **20** | [Schwaiger et al., 1995](../../../../C:%5CUsers%5Ctads%5CDocuments%5CMy%20Writing%5CManuscripts%5CSheep%20QTL%20for%20parasites%5CBenavides%20sheep%20QTL%5Cschwaigeretal1995.pdf) | Scottish Blackface | *Ostertagia circumcincta* | Exon 2 Ovar-DRB1*G2 (MHC Class II) antigen reduces FEC in 22- and 58-fold in September and October, respectively |
| **20** | [Stear et al., 1996](../../../../C:%5CUsers%5Ctads%5CDocuments%5CMy%20Writing%5CManuscripts%5CSheep%20QTL%20for%20parasites%5CBenavides%20sheep%20QTL%5Cstearetal1996.pdf) | Scottish Blackface | *Ostertagia circumcincta* | OLA (MHC Class I) antigen G13br reduced FEC by 10-fold. DRB1 g2 allele also reduces FEC |
| **20** | Riggio et al., 2013 | Scottish Blackface | *Teladorsagia circumcincta* | OAR20_38142478 and SFEC16 |
| **20** | Riggio et al., 2013 | Scottish Blackface | *Teladorsagia circumcincta* | OAR20_42312942 and SFEC16 |
| **20** | Sallé et al., 2012 | Romane × Martinique Black Belly backcross | *Haemonchus contortus* | OAR20_24357620 - OAR20_24626067 and FEC12t |
| **20** | Sallé et al., 2012 | Romane × Martinique Black Belly backcross | *Haemonchus contortus* | s69570 - OAR20_32868803 and FEC34t |
| **21** | [Dominik et al., 2010](../../../../C:%5CUsers%5Ctads%5CDocuments%5CMy%20Writing%5CManuscripts%5CSheep%20QTL%20for%20parasites%5CBenavides%20sheep%20QTL%5CDominiketal2010.pdf) | Romney x Merino backcross | 1st and 2nd *Trichostrongylus colubriformis*, and 3rd *Haemonchus contortus* infection | CSRD272 - BMS1948 and eosinophil change (primary infection) |
| **22** | [Dominik et al., 2010](../../../../C:%5CUsers%5Ctads%5CDocuments%5CMy%20Writing%5CManuscripts%5CSheep%20QTL%20for%20parasites%5CBenavides%20sheep%20QTL%5CDominiketal2010.pdf) | Romney x Merino backcross | 1st and 2nd *Trichostrongylus colubriformis*, and 3rd *Haemonchus contortus* infection | HEL11 - BMS6041 and FWEC (secondary infection) |
| **22** | [Silva et al., 2011](../../../../C:%5CUsers%5Ctads%5CDocuments%5CMy%20Writing%5CManuscripts%5CSheep%20QTL%20for%20parasites%5CBenavides%20sheep%20QTL%5CSilvaetal2010.pdf) | Red Maasai x Dorper backcross | *Haemonchus contortus* and *Trichostrongylus colubriformis* | Significant QTL for AVFEC at Chr 22 markers at 41.0 cM position and for AVPCV and PCVD at 40.0 cM and 41.0 cM positions, respectively |
| **23** | [Crawford et al., 2006](../../../../C:%5CUsers%5Ctads%5CDocuments%5CMy%20Writing%5CManuscripts%5CSheep%20QTL%20for%20parasites%5CBenavides%20sheep%20QTL%5Ccrawfordetal2006.pdf) | Romney x Coopworth cross | *Trichostrongyle* and *Nematodirus* | ILSTS65 - McMAI/ADCYCAPI and ELISA4 (sire154) |
| **23** | [Crawford et al., 2006](../../../../C:%5CUsers%5Ctads%5CDocuments%5CMy%20Writing%5CManuscripts%5CSheep%20QTL%20for%20parasites%5CBenavides%20sheep%20QTL%5Ccrawfordetal2006.pdf) | Romney x Coopworth cross | *Trichostrongyle* and *Nematodirus* | ILSTS42/BL4 - Centromere/BM226 and LIGE (sire154) |
| **23** | Sallé et al., 2012 | Romane × Martinique Black Belly backcross | *Haemonchus contortus* | OAR23_61434545 - OAR23_61932991 and FEC34t |
| **23** | Sallé et al., 2012 | Romane × Martinique Black Belly backcross | *Haemonchus contortus* | s72843 - OAR23_16996616 and FECt_a |
| **23** | Marshall et al., 2012 | Red Maasai x Dorper backcross | *Haemonchus contortus* and *Trichostrongylus colubriformis* | BMS2526 - BMS2270 and FEC |
| **24** | Riggio et al., 2013 | Scottish Blackface | *Teladorsagia circumcincta* | s40989 and SFEC24 |
| **26** | Marshall et al., 2012 | Red Maasai x Dorper backcross | *Haemonchus contortus* and *Trichostrongylus colubriformis* | CRSD163 and FEC |

References

1. Arranz JJ, Pérez J, El Zarei MF, Gutiérrez-Gil B, de la Fuente LF, Álvarez L, et al. Búsqueda de regiones genómicas con influencia sobre la resistencia a las Trichostrongilidosis en el ganado ovino de la raza Churra. 12a Reunión Nacional de Mejora Genética Animal. Arucas, Gran Canaria, Spain. 2004; pp. 4.

2. Beh KJ, Hulme DJ, Callaghan MJ, Leish Z, Lenane I, Windon RG, et al. A genome scan for quantitative trait loci affecting resistance to *Trichostrongylus colubriformis* in sheep. Animal Genetics. 2002; 33: 97-106.

3. Diez-Tascón C, MacDonald PA, Dodds KG, McEwan JC, Crawford AM. A screen of chromosome 1 for QTL affecting nematode resistance in an ovine outcross population. 7th World Congress on Genetics Applied to Livestock Production. Montpellier, France. 2002; pp. 4.

4. Ellis NA, Kayis SA, Fullard KJ, Townley DJ, Khatkar D, Attard G, et al. Targeted mapping of QTL on chromosomes 1 and 3 for parasite resistance in sheep. Proceedings of the Association for the Advancement of Animal Breeding and Genetics. Barossa Valley, SA, Australia. 2009; 18. pp. 4.

5. Gutiérrez-Gil B, Peréz J, Álvarez L, Martinez-Valladares M, de la Fuente LF, Bayón Y, et al. Quantitative trait loci for resistance to trichostrongylid infection in Spanish Churra sheep. Genetics Selection Evolution. 2009; 41: 46.

6. Marshall K, Maddox JF, Lee SH, Zhang Y, Kahn L, Graser HU, et al. Genetic mapping of quantitative trait loci for resistance to *Haemonchus contortus* in sheep. Animal Genetics. 2009; 40: 262-272.

7. Riggio V, Matika O, Pong-Wong R, Stear MJ, Bishop SC. Genome-wide association and regional heritability mapping to identify loci underlying variation in nematode resistance and body weight in Scottish Blackface lambs. Heredity. 2013; 110: 420-429.

8. Crawford A, Paterson K, Dodds K, Diez-Tascón C, Williamson P, Thompson MR, et al. Discovery of quantitative trait loci for resistance to parasitic nematode infection in sheep: I. Analysis of outcross pedigrees. BMC Genomics. 2006; 7: 178.

9. Davies G, Stear MJ, Benothman M, Abuagob O, Kerr A, Mitchell S, et al. Quantitative trait loci associated with parasitic infection in Scottish Blackface sheep. Heredity. 2006; 96: 252-258.

10. Marshall K, Mugambi J, Nagda S, Sonstegard T, Van Tassell C, Baker RL, et al. Quantitative trait loci for resistance to *Haemonchus contortus* artificial challenge in Red Maasai and Dorper sheep of East Africa. Animal Genetics. 2012; 44: 285-295.

11. Beraldi D, McRae AF, Gratten J, Pilkington JG, Slate J, Visscher PM, et al. Quantitative trait loci (QTL) mapping of resistance to strongyles and coccidia in the free-living Soay sheep (*Ovis aries*). International Journal for Parasitology. 2007; 37: 121-129.

12. Coltman DW, Wilson K, Pilkington JG, Stear MJ, Pemberton JM. A microsatellite polymorphism in the gamma interferon gene is associated with resistance to gastrointestinal nematodes in a naturally-parasitized population of Soay sheep. Parasitology. 2001; 122: 571-582.

13. Crawford AM, McEwan JC, Doods KG, Wright CS, Bisset SA, Macdonald PA, et al. Resistance to nematode parasites in sheep: how important are the MHC genes? Proceeding of the Association for the Advancement of Animal Breeding and Genetics. Dubbo, NSW, Australia. 1997; pp. 5.

14. Dominik S, Hunt PW, McNally J, Murrell A, Hall A, Purvis IW*.* Detection of quantitative trait loci for internal parasite resistance in sheep. I. Linkage analysis in a Romney x Merino sheep backcross population. Parasitology. 2010; 137: 1275-1282.

15. Moreno CR, Gruner L, Scala A, Mura L, Schibler L, Amigues Y, et al. QTL for resistance to internal parasites in two designs based on natural and experimental conditions of infection. 8th World Conference on Genetics Applied to Livestock Production. Belo Horizonte, MG, Brazil. 2006; pp. 4.

16. Paterson S, Wilson K, Pemberton JM. Major histocompatibility complex variation associated with juvenile survival and parasite resistance in a large unmanaged ungulate population (*Ovis aries* L.). Proceedings of the National Academy of Sciences of the United States of America. 1998; 95: 3714-3719.

17. Paterson KA, McEwan JC, Dodds KG, Crawford AM. Fine mapping a locus affecting host resistance to internal parasites in sheep. Proceedings of the Association for the Advancement of Animal Breeding and Genetics. Queenstown, New Zealand. 2001; pp. 3.

18. Sayers G, Good B, Hanrahan JP, Ryan M, Sweeney T. Intron 1 of the interferon [gamma] gene: Its role in nematode resistance in Suffolk and Texel sheep breeds. Research in Veterinary Science. 2005; 79: 191-196.

19. Silva M, Sonstegard T, Hanotte O, Mugambi J, Garcia J, Nagda S, et al. Identification of quantitative trait loci affecting resistance to gastrointestinal parasites in a double backcross population of Red Maasai and Dorper sheep. Animal Genetics. 2011; 43: 63-71.

20. Benavides MV, Weimer TA, Borba MFS, Berne MEA, Sacco AMS. Association between microsatellite markers of sheep chromosome 5 and faecal egg counts. Small Ruminant Research. 2002; 46: 97-105.

21. Benavides MV, Weimer TA, Borba MFS, Berne MEA, Sacco AMS. Genetic analyses of polymorphisms on ovine chromosomes 5 and 20 and their effect on resistance to internal parasites. Small Ruminant Research. 2009; 83: 67-73.

22. Sallé G, Jacquiet P, Gruner L, Cortet J, Sauvé C, Prévot F, et al. A genome scan for QTL affecting resistance to *Haemonchus contortus* in sheep. Journal of Animal Science. 2012; 90: 4690-4705.

23. Clarke RA, Burn AL, Lenane I, Windon RG, Beh KJ. Molecular analysis and nematode resistance association of a polymorphism at the 5' end of the sheep IgE gene. Veterinary Immunology and Immunopathology. 2001; 79: 15-29.

24. Buitkamp J, Filmether P, Stear MJ, Epplen JT. Class I and class II major histocompatibility complex alleles are associated with faecal egg counts following natural, predominantly *Ostertagia circumcincta* infection. Parasitology Research. 1996; 82: 693-696.

25. Charon K. Genes controlling resistance to gastrointestinal nematode in ruminants. Animal Science Papers and Reports. 2004; 22: 5.

26. Douch PGC, Outteridge PM. The relationship between ovine lymphocyte antigens and parasitological and production parameters in Romney sheep. International Journal for Parasitology. 1989; 19: 35-41.

27. Janßen M, Weimann C, Gauly M, Erhardt G. Associations between infections with *Haemonchus contortus* and genetic markers on ovine chromosome 20. 7th World Congress on Genetics Applied to Livestock Production. Montpellier, France. 2002; pp. 4.

28. Janßen M, Weimann C, Brandt H, Gauly M, Erhardt G. Parasitological parameters after artificial infections with *Haemonchus contortus* in Merinoland sheep and its association to genetic markers on chromosome 20. Archiv für Tierzucht. Göttingen, Germany. 2004; pp. 36-42.

29. Keane OM, Dodds KG, Crawford AM, McEwan JC. Transcriptional profiling of *Ovis aries* identifies Ovar-DQA1 allele frequency differences between nematode-resistant and susceptible selection lines. Physiological Genomics. 2007; 30: 253-261.

30. Luffau G, Khang J, Bouix J, Nguyen T, Cullen P, Ricordeau G, et al. Resistance to experimental infections with *Haemonchus contortus* in Romanov sheep. Genetics Selection Evolution. 1990; 22: 205 - 229.

31. Outteridge PM, Jones WO, Edgar JA. The use of fluorescent probes as markers for sheep lymphocyte subpopulations. Veterinary Immunology and Immunopathology. 1988; 19: 141-151.

32. Sayers G, Good B, Hanrahan JP, Ryan M, Angles JM, Sweeney T. Major Histocompatibility Complex DRB1 gene: its role in nematode resistance in Suffolk and Texel sheep breeds. Parasitology. 2005; 131: 403-409.

33. Schwaiger FW, Gostomski D, Stear MJ, Duncan JL, McKellar QA, Epplen JT, et al. An ovine major histocompatibility complex DRB1 allele is associated with low faecal egg counts following natural, predominantly *Ostertagia circumcincta* infection. International Journal for Parasitology. 1995; 25: 815-822.

34. Stear MJ, Bairden K, Bishop SC, Buitkamp J, Epplen JT, Gostomski D, et al. An ovine lymphocyte antigen is associated with reduced faecal egg counts in four-month-old lambs following natural, predominantly *Ostertagia circumcincta* infection. International Journal for Parasitology. 1996; 26: 423-428.
